# Characterization of the *Runx* Gene Family in a Jawless Vertebrate, the Japanese Lamprey (*Lethenteron japonicum*)

**DOI:** 10.1371/journal.pone.0113445

**Published:** 2014-11-18

**Authors:** Giselle Sek Suan Nah, Boon-Hui Tay, Sydney Brenner, Motomi Osato, Byrappa Venkatesh

**Affiliations:** 1 Institute of Molecular and Cell Biology, Agency for Science, Technology and Research (A*STAR), Singapore, Singapore; 2 Okinawa Institute of Science and Technology Graduate University, Onna-son, Okinawa 904-0495, Japan; 3 Cancer Science Institute of Singapore, National University of Singapore, Singapore, Singapore; 4 Institute of Bioengineering and Nanotechnology, Agency for Science, Technology and Research, Singapore, Singapore; 5 Department of Paediatrics, Yong Loo Lin School of Medicine, National University of Singapore, Singapore, Singapore; California State University Fullerton, United States of America

## Abstract

The cyclostomes (jawless vertebrates), comprising lampreys and hagfishes, are the sister group of jawed vertebrates (gnathostomes) and are hence an important group for the study of vertebrate evolution. In mammals, three *Runx* genes, *Runx1*, *Runx2* and *Runx3*, encode transcription factors that are essential for cell proliferation and differentiation in major developmental pathways such as haematopoiesis, skeletogenesis and neurogenesis and are frequently associated with diseases. We describe here the characterization of *Runx* gene family members from a cyclostome, the Japanese lamprey (*Lethenteron japonicum*). The Japanese lamprey contains three *Runx* genes, *RunxA*, *RunxB*, and *RunxC*. However, phylogenetic and synteny analyses suggest that they are not one-to-one orthologs of gnathostome *Runx1*, *Runx2* and *Runx3*. The major protein domains and motifs found in gnathostome Runx proteins are highly conserved in the lamprey Runx proteins. Although all gnathostome *Runx* genes each contain two alternative promoters, P1 (distal) and P2 (proximal), only lamprey *RunxB* possesses the alternative promoters; lamprey *RunxA* and *RunxC* contain only P2 and P1 promoter, respectively. Furthermore, the three lamprey *Runx* genes give rise to fewer alternative isoforms than the three gnathostome *Runx* genes. The promoters of the lamprey *Runx* genes lack the tandem Runx-binding motifs that are highly conserved among the P1 promoters of gnathostome *Runx1*, *Runx2* and *Runx3* genes; instead these promoters contain dispersed single Runx-binding motifs. The 3′UTR of lamprey *RunxB* contains binding sites for miR-27 and miR-130b/301ab, which are conserved in mammalian *Runx1* and *Runx3*, respectively. Overall, the *Runx* genes in lamprey seem to have experienced a different evolutionary trajectory from that of gnathostome *Runx* genes which are highly conserved all the way from cartilaginous fishes to mammals.

## Introduction

The polyomavirus enhancer-binding protein 2 (PEBP2) or core-binding factor (CBF) is an ancient Runt domain heterodimeric transcription factor of α and β subunits. In human, the α-subunit is encoded by three *Runt* gene family members, *RUNX1*, *RUNX2* and *RUNX3* that contain an evolutionarily conserved 128 amino acid Runt domain responsible for DNA binding and heterodimerization with the β-subunit. The β-subunit comprises a single protein, RUNXβ (also known as PEBP2β or CBFβ) that does not directly interact with DNA but serves to allosterically enhance the DNA-binding activity of the α-subunit and regulate its turnover by protecting it from ubiquitin-proteasome-mediated degradation [1], [2]. Basally branching chordates such as amphioxus (*Branchiostoma floridae*) and tunicates (*Ciona* and *Oikopleura*) possess a single *Runx* gene. By contrast, vertebrates such as cartilaginous fishes (e.g. elephant shark, dogfish) and tetrapods contain three *Runx* genes (*Runx1*, *Runx2* and *Runx3*) [3], [4], [5] owing to the two rounds of whole genome duplication at the base of vertebrates [6], [7]. Teleost fishes such as zebrafish contain a duplicated *Runx2* gene (*Runx2b*) that resulted from an additional teleost-specific genome duplication event [8]. On the other hand, pufferfishes (fugu and *Tetraodon*) possess three *Runx* genes like tetrapods and an additional fourth *Runx* gene called *frRunt* [9], [10], which is hypothesized to represent an ancestral vertebrate *Runx* gene that was lost in tetrapods and zebrafish [9]. In contrast to multiple copies of α-subunit encoding *Runx* genes, *Runxb* is present as a single copy in vertebrates as well as in invertebrates analysed, with the exception of the fruit fly (*Drosophila melanogaster*) that possesses two orthologs of *Runxβ* genes, *Brother* and *Big brother* [11], [12], [13].

In mammals, the three *Runx* genes play pivotal roles in several developmental processes including haematopoiesis, neurogenesis, and skeletogenesis. *Runx1* is required for the emergence and maintenance of hematopoietic stem cells [14], [15] and is frequently mutated in human leukaemia [16], [17], [18]. *Runx2* is indispensable for osteogenesis as evidenced by the complete lack of ossified skeleton in *Runx2*-deficient mice [19]. Haploinsufficiency of *RUNX2* results in the autosomal dominant bone disease, cleidocranial dysplasia [20]. *Runx3* is involved in diverse biological pathways including the regulation of epithelial homeostasis in the gastrointestinal tract [21], [22], development of T cells during thymopoiesis [23] and the differentiation of various cell types of the immune system [24], [25], [26]. *Runx3* is also essential for the differentiation of proprioceptive neurons [27] and chondrocyte maturation during skeletogenesis [28].

The living vertebrates are divided into two broad groups: the jawless vertebrates (cyclostomes) and jawed vertebrates (gnathostomes). While the gnathostomes are represented by cartilaginous fishes, ray-finned fishes, lobe-finned fishes and tetrapods, the cyclostomes are represented by only lampreys and hagfish which constitute a monophyletic group. Cyclostomes diverged from gnathostomes ∼500 Mya and are morphologically and physiologically quite distinct from gnathostomes. They possess a single median dorsal “nostril” as opposed to ventrally located nostrils in gnathostomes. In addition, they lack mineralized tissues, hinged jaws, paired appendages, pancreas and spleen [29]. Although cyclostomes lack an immunoglobulin-based adaptive immune system, a thymus-like organ called the ‘thymoid’, has been identified at the tips of gill filaments in the gill basket [30] and is the site of antigen receptor gene assembly of T-cell like lymphocytes [31]. The adaptive immune system of cyclostomes makes use of an alternative immune receptor system different from that of gnathostomes. Instead of T-cell and B-cell receptors generated from the immunoglobulin superfamily, cyclostomes utilize variable lymphocyte receptors (VLR) that are assembled from leucine-rich repeat modules (reviewed in [32]). These distinctive morphological and physiological traits along with the unique phylogenetic position of cyclostomes make them an important group for understanding the evolution and function of vertebrate *Runx* genes.

Full-length coding sequences of two *Runx* genes, designated *MgRunxA* and *MgRunxB* have been previously cloned in a cyclostome, the Atlantic hagfish (*Myxine glutinosa*). These *Runx* genes were found to be expressed in cartilaginous tissues, suggestive of their involvement in early vertebrate skeletogenesis [4]. Mining of the genome assembly of the sea lamprey (*Petromyzon marinus*) led to the identification of partial sequences for only two *Runx* genes, *PmRunxA* and *PmRunxB* and the conclusion that this is likely to be the full complement of *Runx* genes in lamprey [33]. However, it should be noted that the sea lamprey genome assembly was generated from DNA extracted from a somatic tissue (liver) that has been shown to lose ∼20% of genomic DNA due to developmentally programmed rearrangement during early embryonic development [34] and hence does not contain the full complement of genes present in the germline genome. Recently, the whole genome sequence of another lamprey, the Japanese lamprey (*Lethenteron japonicum*) was generated using DNA from the testis [35] (http://jlampreygenome.imcb.a-star.edu.sg/). The Japanese lamprey and sea lamprey are northern hemisphere lampreys that diverged from each other 30–10 Mya ago [36]. Compared to the 2.3 Gb genome of the sea lamprey, the Japanese lamprey has a relatively smaller genome of ∼1.6 Gb [37]. To improve our understanding of the evolution, function and regulation of *Runx* genes, we mined the Japanese lamprey genome assembly for *Runx* genes and completed the coding sequences for *Runx* genes by RT-PCR and RACE. Our analyses show that the Japanese lamprey genome codes for three *Runx* genes like gnathostomes but they are not the exact one-to-one orthologs of gnathostome *Runx1*, *Runx2* and *Runx3*.

## Materials and Methods

### Ethics statement

The Japanese lamprey specimens were collected from commercial fishermen who routinely catch them like other commercial fishes in Ishikari River near Ebetsu in Hokkaido, Japan. The procedure for extraction of DNA and RNA from lamprey tissues was approved by the Institutional Animal Care and Use Committee of the Biological Resource Centre, Agency for Science, Technology and Research (A*STAR), Singapore.

### Identification of *Runx* genes in the Japanese lamprey genome assembly

The genome assembly of the Japanese lamprey (http://jlampreygenome.imcb.a-star.edu.sg/) was searched with human RUNX1, RUNX2, RUNX3 and RUNXβ protein sequences using the ‘TBLASTN’ algorithm. Scaffolds #47, #165, #769 and #850 containing sequences homologous to human and elephant shark Runx1, Runx2, Runx3 and Runxβ protein sequences were searched against the non-redundant protein database at NCBI using BlastX to identify the coding exons of lamprey *Runx* genes. Missing exons in the scaffolds (presumably due to gaps in the scaffolds) were identified by sequencing RACE (Rapid Amplification of cDNA Ends) products as described below.

### Full-length cDNA cloning by RACE

Total RNA was extracted from various tissues of adult Japanese lamprey using Trizol reagent (Gibco BRL, Grand Island, NY) according to manufacturer's protocol. One µg of total RNA was reverse transcribed into 5′RACE-ready or 3′RACE-ready single-strand cDNA by using the SMART RACE cDNA Amplification kit (Clontech, Palo Alto, CA). Primers were designed for representative exons identified in the Japanese lamprey scaffolds and full-length *Runx* transcripts were obtained by 5′ RACE and/or 3′ RACE (primer sequences available upon request). 5′ and 3′ RACE were each performed in a nested PCR. The first round of PCR was performed with the Universal Primer and a gene-specific primer. The resulting PCR product was diluted 20× and 1 µl used for the nested PCR with the Nested Universal Primer and a second gene-specific primer. All RACE products were cloned into the pGEM-T Easy Vector (Promega, USA), and sequenced completely using the BigDye Terminator Cycle Sequencing Kit (Applied Biosystems, USA) on an ABI 3730×l capillary sequencer (Applied Biosystems, USA). The genome structures of the Japanese lamprey *Runx* genes, including the exon-intron structures, UTRs and transcription start sites were deciphered by mapping the cloned full length cDNA sequences to the Japanese lamprey genomic sequence. Sequences for various isoforms of the Japanese lamprey *Runx* genes have been deposited in GenBank (KJ787775–KJ787788).

### Amino acid alignment and phylogenetic analysis

The full-length protein sequences for human and other chordate *Runx* genes, including the two Runx sequences (RunxA and RunxB) from the Atlantic hagfish were retrieved from the National Center for Biotechnology Information (NCBI) database. RunxA and RunxB from the sea lamprey were not used as they are partial sequences. Multiple sequence alignments were generated using MUSCLE (www.ebi.ac.uk/Tools/msa/muscle/). For phylogenetic analysis, the alignments were trimmed using the Gblocks Server (ver. 0.91b) with less stringent selection parameters [38]. MEGA 6.06 (http://www.megasoftware.net/) was used to determine the most appropriate amino acid substitution model for each dataset. Maximum Likelihood (ML) and Bayesian (BI) methods were used for phylogenetic analyses employing the JTT+G+I or JTT+G model. MEGA 6.06 was used for ML analyses and 100 bootstrap replicates were used for node support. BI analyses were carried out using MrBayes 3.2 (http://mrbayes.csit.fsu.edu/). Two independent runs starting from different random trees were run for 1 million generations with sampling done every 100 generations. A consensus tree was built from all sampled trees excluding the first 2,500 trees (burn-in).

### Synteny analysis

The order of genes in the *Runx* loci of human, chicken, zebrafish, sea anemone (*Nematostella vectensis*) and sponge (*Amphimedon queenslandica*) were obtained from Ensembl (www.ensembl.org); amphioxus (*Branchiostoma floridae*) from the UCSC Genome Browser (http://genome.ucsc.edu/); elephant shark from the elephant shark genome assembly [39] (http://esharkgenome.imcb.astar.edu.sg/) and Japanese lamprey from the Japanese lamprey genome assembly (http://jlampreygenome.imcb.a-star.edu.sg/).

### Expression profiling by qRT-PCR

Purified total RNA was reverse-transcribed into cDNA with Superscript II (Invitrogen, Carlsbad, CA). The single strand cDNA was used as a template in qRT-PCR reactions with KAPA SYBR FAST qPCR Kit reagents (KAPA Biosystems, Boston, MA). Sequences of primers used in qRT-PCR are given in Table S1. All primer pairs span at least one intron which helps to distinguish cDNA sequences from genomic DNA products. Expression levels of *Runx* genes were normalized using *beta-actin* gene as the reference. Quantification of gene expression levels was performed using the comparative *C*_T_ method [40]. The relative expression levels of each *Runx* gene in different tissues were estimated in relation to a reference tissue (one that showed the lowest level of expression among the tissues analysed). Note, however, that these relative expression levels are not comparable between different *Runx* genes.

## Results and Discussion

### Cloning and orthology of Japanese lamprey α-subunit *Runx* family members

**Figure 1:**
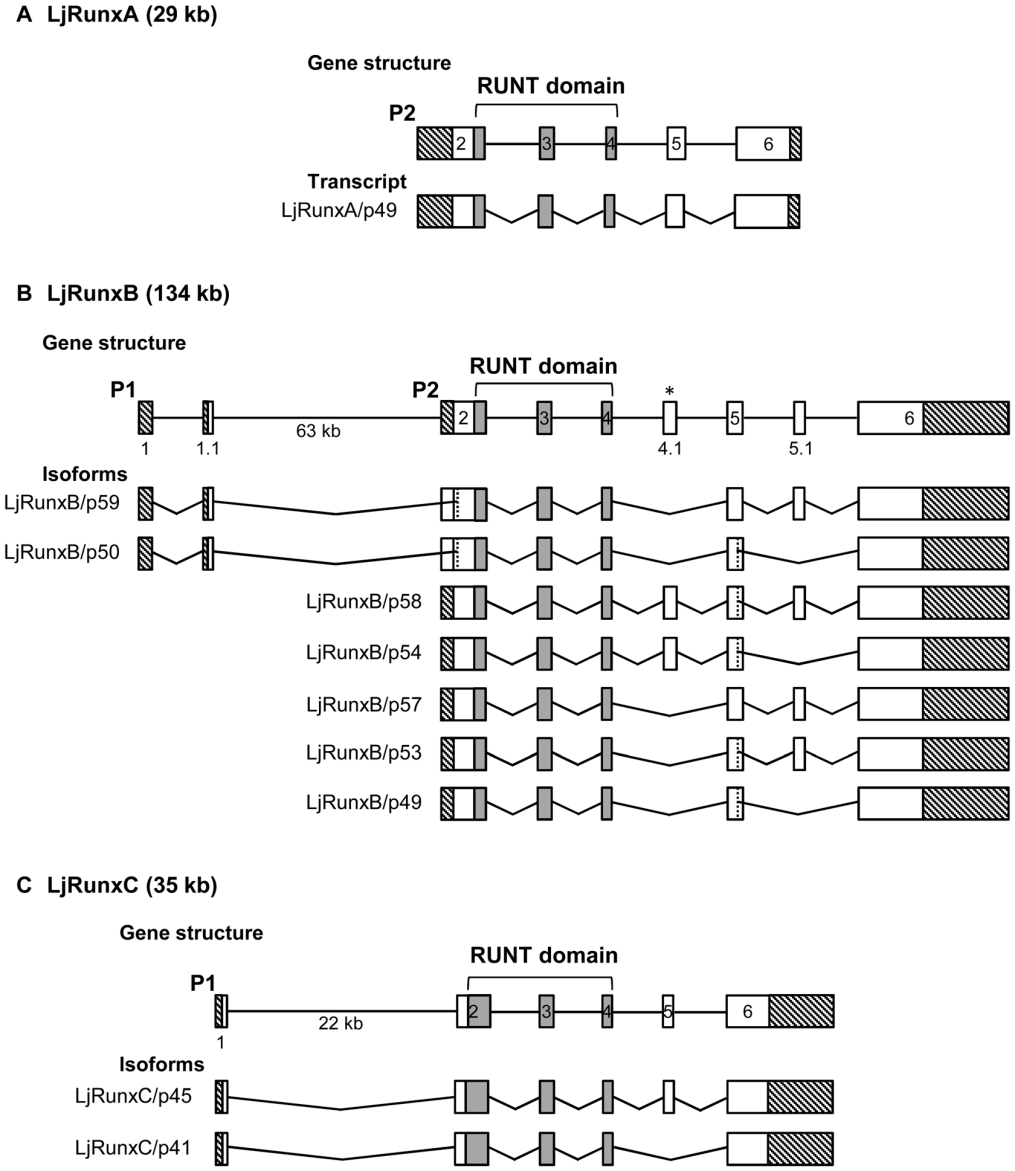
Exon-intron organization of lamprey (*Lj*) *Runx* genes. Schematic representation of the gene structures and transcript isoforms of (A) *LjRunxA*, (B) *LjRunxB* and (C) *LjRunxC*. Exons are indicated by boxes. The vertical dashed lines indicate internal splice sites located within the coding exon. Exons constituting the Runt domain are indicated in grey. The two alternative promoters are denoted as P1 and P2. Crosshatched boxes indicate 5′- and 3′-UTRs. The asterisk (*) indicates an exon in *LjRunxB* that is absent in gnathostome *Runx*. Not drawn to scale.

To identify *Runx* genes in the Japanese lamprey, we BLAST-searched the Japanese lamprey genome assembly [35] using human RUNX protein sequences. We identified three scaffolds (Scaffold_850, 47 and 769) that contained fragments of *Runx* genes. The missing exons and full-length coding sequences of these genes were identified by RT-PCR and/or RACE using cDNA from gill, intestine or skin (Fig. 1 and S1).

**Figure 2:**
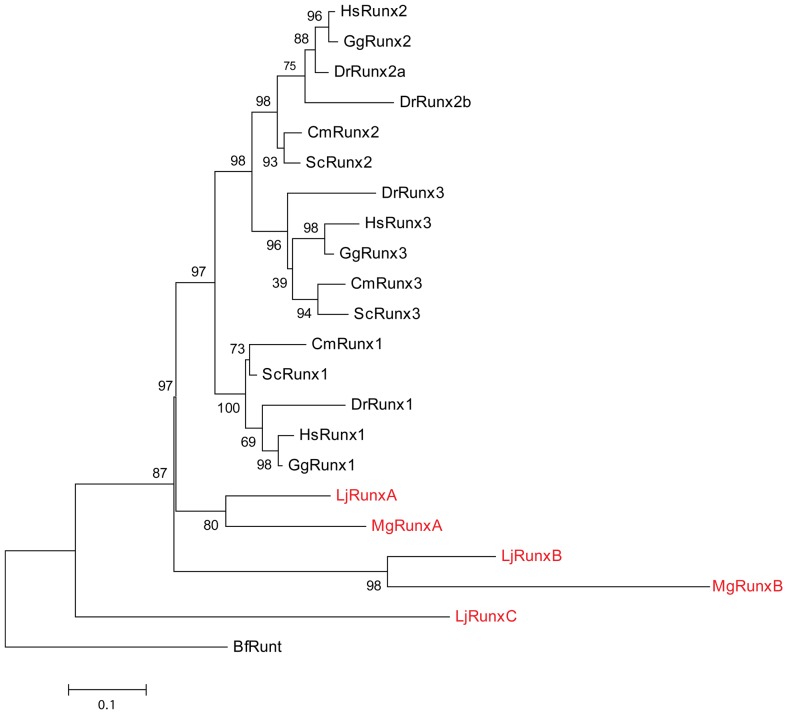
Phylogenetic analysis of chordate Runx sequences. Protein sequences of Japanese lamprey *Runx* genes were aligned with homologous sequences from selected chordates. The gaps in the alignments were trimmed using the Gblocks Server. The resulting protein alignment used for phylogenetic analyses is provided in Fig. S3. Maximum Likelihood (ML) trees were generated for the alignments. Statistical support values for the nodes are shown as ML bootstrap percentages. Hagfish and Japanese lamprey Runx proteins are highlighted in red. Lancelet (*Branchiostoma floridae*) Runt (BfRunt) was used as the outgroup. Hs, *Homo sapiens*; Gg, *Gallus gallus*; Dr, *Danio rerio*; Cm, *Callorhinchus milii*; Sc, *Scyliorhinus canicula*; Mg, *Myxine glutinosa*; Lj, *Lethenteron japonicum*.

To determine the relationships of the three lamprey *Runx* genes to *Runx1*, *Runx2* and *Runx3* of gnathostomes, we carried out phylogenetic analysis using two different algorithms, ML and BI. The *Runt* gene from amphioxus was used as the outgroup. Both ML and BI analyses showed that the Japanese lamprey *RunxA* and *RunxB* genes are orthologs of the hagfish *RunxA* and *RunxB* genes, respectively (Fig. 2 and S2). However, the three lamprey and two hagfish genes were found to cluster outside the three gnathostome genes. In the ML tree, cyclostome *RunxA* formed a sister clade to all three gnathostome *Runx* genes while *RunxB* and *RunxC* genes were represented on separate branches outside the *RunxA*+gnathostome *Runx* clade (Fig. 2). In BI analysis, the combined clade of cyclostome *RunxA* and *RunxB* genes constituted the sister group to all three gnathostome *Runx* genes with *RunxC* being an outgroup (Fig. S2). This pattern of clustering of gnathostome and cyclostome *Runx* genes suggests that three gnathostome *Runx* genes are the result of duplications in the gnathostome ancestor after it diverged from the cyclostome lineage. This implies that the three lamprey *Runx* genes are not the exact one-to-one orthologs of the three gnathostome *Runx* genes.

In addition, we generated an ML tree that included single *Runx* sequences from two more well characterized invertebrate species, *Ciona intestinalis* an urochordate and *Nematostella vectensis* a cnidarian (Fig. S4 and S5). In this tree, the relationships of gnathostome *Runx* genes are essentially similar to that depicted in Fig. 2, and the cyclostome genes cluster outside the gnathostome clade. However, some differences in topology are observed among the cyclostome branches (Fig. S5). These branches, though, are not well supported (bootstrap values of 40 and 37), suggesting that these relationships are not reliable. We believe that such changes to the topology are due to the highly divergent sequences of *Ciona* and *Nematostella* (see alignment in Fig. S4).

**Figure 3:**
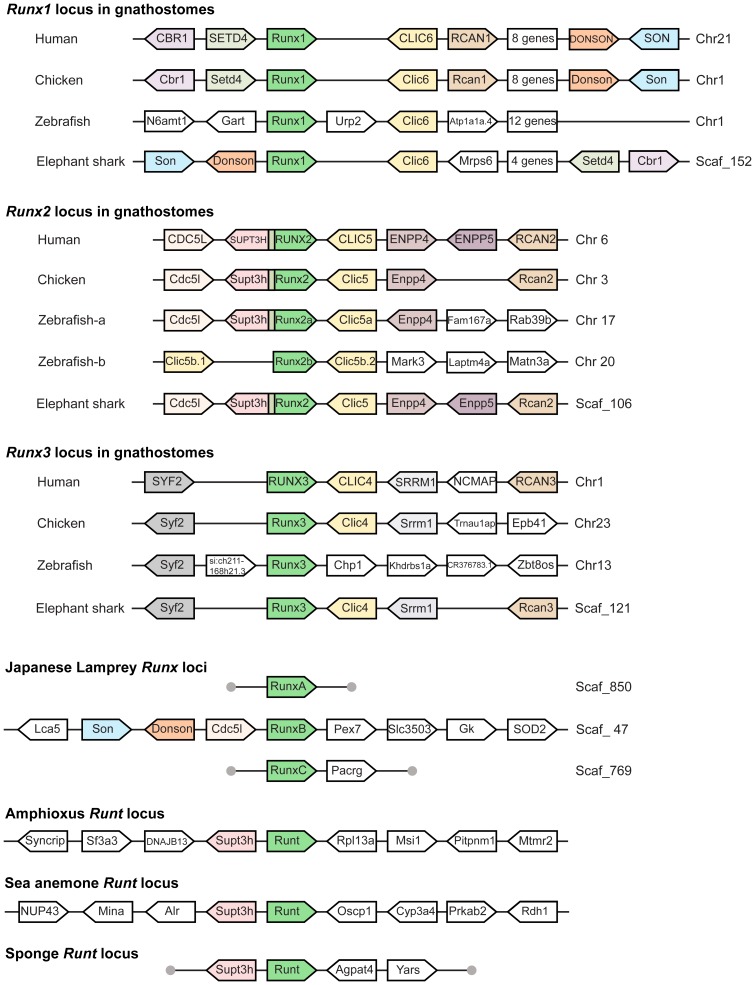
Synteny of genes in the *Runx* loci of Japanese lamprey and selected metazoans. Genes are represented by block arrows. Genes with conserved synteny are coloured. Clusters of some non-syntenic genes are represented as white boxes. Grey circles indicate the end of scaffolds.

The three *Runx* genes in the gnathostomes are likely to be the result of two rounds of whole genome duplication (2R) that occurred early during the evolution of vertebrates [6], [41], [42]. Consistent with this hypothesis, synteny of sets of paralogous genes are conserved in the gnathostome *Runx1*, *Runx2* and *Runx3* gene loci. For example, synteny of paralogs of *Runx*, *Clic* and *Rcan* are conserved in the *Runx1*, *Runx2* and *Runx3* loci of all gnathostomes analysed so far (Fig. 3). Conserved synteny of blocks of genes between genomes provides additional support for the orthologous relationship of genes in the syntenic block. For instance, orthologs of several genes in the human *RUNX2* locus (e.g. *CDC5L*, *SUPT3H*, *CLIC5*) are conserved in the *Runx2* locus of chicken, zebrafish and elephant shark indicating that the *Runx* gene in this locus of these gnathostomes is indeed an ortholog of human *RUNX2* (Fig. 3). An interesting pair of syntenic genes in the gnathostome *Runx2* locus is the tightly linked *RUNX2* and *SUPT3H* genes whose first exons overlap. The intertwined organization of these two genes is conserved in all gnathostomes examined, with the exception of the duplicate locus of zebrafish *Runx2b* in which the duplicate *Supt3hb* gene has been lost (Fig. 3). The close linkage between *Runx* and *Supt3h* genes seems to be an ancestral state, because in invertebrates such as amphioxus, sea anemone (*Nematostella vectensis*) and demosponge (*Amphimedon queenslandica*), the *Supt3h* gene is located next to the single *Runt* gene (Fig. 3). It can therefore be inferred that after the duplication of the ancestral vertebrate *Runx* locus in the gnathostome ancestor, the paralogs of *Supt3h* linked to *Runx1* and *Runx3* loci were lost whereas the paralog in *Runx2* locus was retained.

In order to verify the orthology relationships of the three lamprey *Runx* genes to gnathostome *Runx* genes, we analysed the synteny of genes at the lamprey *Runx* gene loci. Interestingly, none of the lamprey *Runx* genes is linked to a *Supt3h* gene. A TBLASTN search of the Japanese lamprey genome assembly using human and elephant shark Supt3h protein sequences identified a single *Supt3h* gene located on a scaffold (Scaf_1) distinct from those containing *Runx* genes. Either this gene previously located next to a *Runx* gene was translocated to a new location in the lamprey genome or the *Runx* gene linked to it has been lost. Of the three Japanese lamprey *Runx* genes, the most extensive synteny information is available for *RunxB*. The syntenic genes of the lamprey *RunxB* include three genes (*Son*, *Donson* and *Cdc5l*) whose orthologs are conserved in the *Runx* loci of gnathostomes. However, while the ortholog of *Son* and *Donson* are found in the gnathostome *Runx1* locus, the ortholog of *Cdc5l* is found linked to gnathostome *Runx2*. The synteny of genes in the lamprey *RunxB* locus therefore suggests that *RunxB* is not an exact ortholog of either *Runx1* or *Runx2* of gnathostomes. This provides further support to the inference of the phylogenetic analysis that the three lamprey *Runx* genes are not one-to-one orthologs of the three gnathostome *Runx* genes.

### Genomic organization of Japanese lamprey α-subunit *Runx* genes

The exon-intron organization of the three gnathostome *Runx* genes is largely similar except for *Runx3* which characteristically lacks an exon (exon 5.1) at the C-terminal end, that is present in both *Runx1* and *Runx2* (Fig. S1). The three lamprey α-subunit *Runx* genes share several structural similarities with those of gnathostomes, including three exons that encode the highly conserved Runt domain (Fig. 1 and S1). Interestingly, of the three lamprey *Runx* genes, only *RunxB* contains the exon equivalent of exon 5.1 of gnathostome *Runx1* and *Runx2* genes and is therefore structurally more similar to these two gnathostome *Runx* genes. In addition, *RunxB* contains an extra exon (exon 4.1) that is not equivalent to any of the gnathostome exons including exon 4.1 in elephant shark *Runx1* (Fig. 1 and S1). This feature distinguishes the lamprey *RunxB* gene from gnathostome *Runx1* and *Runx2*. Lamprey *RunxA* and *RunxC* both lack the exon equivalent to exon 5.1 and are thus more similar to the gnathostome *Runx3* gene (Fig. 1A, C and S1). Since phylogenetic and synteny analyses do not support an orthology relationship between these two lamprey genes and the gnathostome *Runx3* gene, their similar structure appears to be the result of convergent evolution.

All gnathostome *Runx* genes are transcribed from two alternative promoters, P1 (distal) and P2 (proximal) that are separated by a characteristically large intron (Fig. S1). Of the three lamprey *Runx* genes, only *RunxB* contains both P1 and P2 promoters (Fig. 1). Lamprey *RunxA* contains only the proximal (P2) promoter. Our efforts to identify a distal promoter by 5′RACE using cDNA from several tissues failed to yield transcripts originating from a distal promoter and led us to conclude that the P1 promoter is absent in *RunxA* (Fig. 1A). By contrast, lamprey *RunxC* contains the P1 promoter but lacks the P2 promoter (Fig. 1C). The translation initiation codon (ATG) associated with P2 promoter has been mutated to another codon in this gene and the open reading frame which is devoid of an ATG codon runs all the way to the 5′end of exon 2. Thus, *RunxC* has lost the potential to generate transcripts from the proximal promoter. The presence of two alternative promoters in all gnathostome *Runx* genes and the lamprey *RunxB* gene suggests that the two alternative promoters had already emerged in the single ancestral *Runx* gene in the common ancestor of gnathostomes and cyclostomes. Following the duplication of the ancestral *Runx* gene, lamprey *RunxA* and *RunxC* genes secondarily lost P1 and P2 promoters, respectively.

Each of the gnathostome *Runx* genes express a diverse repertoire of isoforms arising from two alternative promoters (P1 and P2) as well as by alternative splicing of exons (Fig. 1). These isoforms are differentially expressed during development and exert a variety of biological functions [43], [44]. Of the three lamprey *Runx* genes, only *RunxB* has the potential to generate isoforms originating from two alternative promoters. In addition, it gives rise to multiple isoforms resulting from alternative splicing of exons similar to gnathostome *Runx* genes (Fig. 1B). By contrast, we could identify only one transcript for lamprey *RunxA* and two isoforms for *RunxC* (Fig. 1A and C). Thus, these lamprey genes give rise to fewer isoforms than the lamprey *RunxB* gene and the three gnathostome *Runx* genes.

The P1 and P2 promoter regions of gnathostome *Runx* genes harbour binding sites for transcription factors that mediate transcriptional regulation of *Runx* genes. Notably, the P1 promoters of gnathostome *Runx1*, *2* and *3* contain two tandem binding sites for Runx (Pu/TACCPuCA) at similar locations in the 5′UTRs [3]. These highly conserved binding sites have been implicated in the auto and cross-regulation of the three gnathostome *Runx* genes [45], [46]. The presence of these sites in the three gnathostome *Runx* genes suggests that they were present in their single ancestral *Runx* gene. In contrast to gnathostome *Runx* genes, the P1 promoters of the lamprey *RunxB* and *RunxC* genes lack these characteristic tandem Runx binding sites. Instead, the P1 promoter of *RunxB* contains dispersed single Runx binding sites at positions −506, −282 and +131. In addition, its P2 promoter contains one Runx binding site at position −328 (data not shown). However, no such single Runx binding site is found in the P1 promoter of *RunxC* or in the P2 promoter of *RunxA*. It remains to be seen if these dispersed Runx motifs in the lamprey *RunxB* promoters are involved in its auto-regulation and/or in its cross-regulation by RunxA and RunxC.

**Figure 4:**
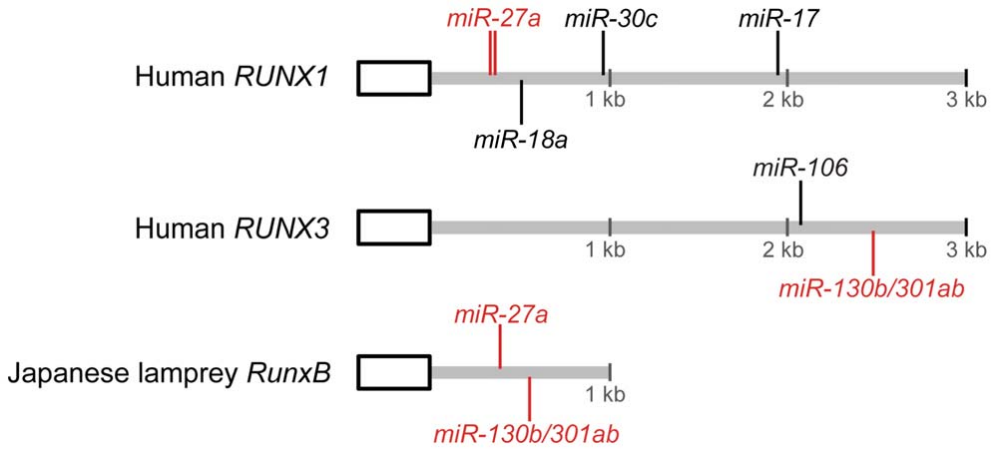
miRNA binding sites in the 3′UTR of human *Runx1* and *Runx3* and Japanese lamprey *RunxB* genes. The last coding region of *Runx* gene is represented by a rectangle and 3′UTR by a grey line. Positions of miRNA binding sites are indicated by vertical lines. Binding sites conserved in human and Japanese lamprey are shown in red.

The expression of gnathostome *Runx1–3* genes is post-transcriptionally regulated by miRNAs that target highly conserved miRNA binding sites in the 3′UTR of their transcripts [47], [48], [49]. To verify if the lamprey *Runx* genes are also regulated by miRNAs, we searched the 3′UTRs of the lamprey *Runx* genes for seed miRNA binding sites that are well characterized in the mammalian *Runx* genes. While no such binding sites were found in *RunxA* and *RunxC*, *RunxB* was found to contain binding sites for miR-27 and miR-130b/301ab (Fig. 4). In mammals, the binding site for miR-27 is present in *Runx1* whereas that for miR-130b/301ab is present in *Runx3* (Fig. 4). It is therefore possible that the single ancestral vertebrate *Runx* gene contained sites for both miRNAs and that they were differentially lost in mammalian *Runx1* and *Runx3* genes. Murine miR-27 has been shown to be involved in the regulation of *Runx1* in megakaryocytic and granulocytic differentiation [48] whereas human miR-130b and miR-301a have been implicated in the down-regulation of *RUNX3* in gastric cancer [50], [51]. These three miRNAs have been previously shown to be expressed in cyclostomes such as the sea lamprey and the Atlantic hagfish [52]. It is therefore likely that the predicted binding sites for these miRNAs in Japanese lamprey *RunxB* are functional and mediate regulation of *RunxB*.

### Comparison of Japanese lamprey Runx α-subunit protein sequences

The domain structures and motifs of Runx proteins are highly conserved among gnathostome Runx1–3. These include the 128 amino acid Runt domain that is required for their DNA binding and heterodimerization with the β-subunit, as well as the transactivation domain (TAD) and inhibitory domain (ID) located at their carboxy termini. In addition, gnathostome Runx proteins contain a nuclear localization signal (NLS) located contiguous to the Runt domain that mediates their nuclear import as well as a nuclear matrix targeting signal (NMTS) in the TAD that directs Runx transcription factors to specific nuclear-matrix associated sites involved in the regulation of gene expression. Other distinctive features of gnathostome Runx proteins include the PY and VWPRY motifs that mediate the transcriptional activity of Runx proteins by recruiting different interacting partners. The proline-rich PY motif in the TAD mediates the binding of Runx proteins to WW domain-containing proteins, such as Yes-associated protein (YAP), transcriptional co-activator with PDZ-binding motif (TAZ), and Smurf [53], [54], [55]; while the C-terminal VWRPY motif recruits the Groucho/Transducin-like enhancer (TLE) transcriptional co-repressor for transcriptional repression [56].

**Figure 5:**
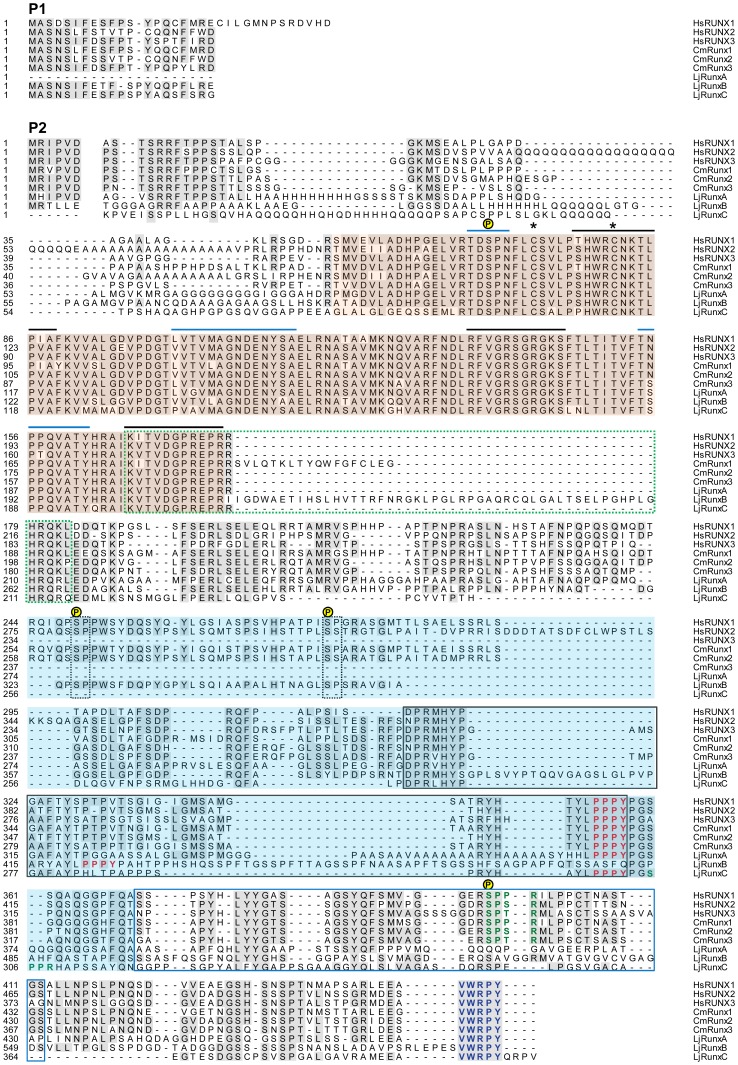
Runx α-subunit proteins in human, elephant shark and Japanese lamprey. Alignment of human, elephant shark and Japanese lamprey Runx α-subunit proteins. The first block shows the amino-terminal part of the protein derived from the P1 promoter that differs from that derived from the P2 promoter. The highly conserved Runt domain is highlighted in pink. Within the Runt domain, surfaces involved in DNA contact and interaction with the β-subunit are denoted by black and blue lines, respectively. Cysteine residues involved in the redox regulation of DNA-binding activity are indicated with asterisks. Nuclear localization signal (NLS) is demarcated by a green dashed box. The PY and VWRPY motifs are indicated in red and blue, respectively. The transactivation domain (TAD) is highlighted in blue and the inhibitory domain (ID) is boxed in blue. The nuclear matrix targeting signal (NMTS) is boxed in black. Minimal consensus sequences for phosphorylation by Erk are boxed by dashed black lines. The consensus phosphorylation site for Cdc2 is indicated in green. The residue targeted for phosophorylation is indicated by ℗. Hs, *Homo sapiens*; Cm, *Callorhinchus milii*; Lj, *Lethenteron japonicum*.

All of these domains and motifs are conserved in the Japanese lamprey RunxA, B and C proteins (Fig. 5). All three proteins contain the characteristic Runt domain, within which, residues for DNA-binding and interaction with the β-subunit [57], [58] are remarkably well conserved with those of gnathostome Runx proteins. The NLS, NMTS, PY and VWPRY motifs are also conserved in lamprey Runx proteins albeit with slight variations: while gnathostome Runx proteins terminate at the VWPRY motif, the open reading frame of lamprey RunxC continues beyond the VWPRY motif to include four additional amino acids (Fig. 5). Among all known Runx proteins, this peculiar feature is seen only in the single Runt protein of *Ciona*. The biological significance of these extra residues is unknown. Additionally, lamprey RunxB contains a unique stretch of 47 amino acids within the NLS domain of RunxB (Fig. 5, boxed in green dotted lines). This sequence is encoded by the lamprey-specific extra exon 4.1 that is alternatively spliced in two of the isoforms of *RunxB* (Fig. 1B). Whether these intervening amino acids affect the nuclear translocation of these RunxB isoforms remains to be verified. The N-termini of the lamprey Runx P1 isoforms beginning with MAS(N/D)S are highly conserved with gnathostome Runx proteins. However, the N-termini of the P2 isoforms of the lamprey proteins vary from those of gnathostomes which comprise MR(I/V)PV. The N-terminal sequences of the lamprey RunxA and RunxB P2 isoforms contain MHIPV and MRTLL, respectively. Similar divergent N-terminal sequences have also been noted in the P2 promoters of *Runx1* (MVFLW), *Runx2* (MRPIV) and *Runx3* (MHIPV) genes of teleost fishes such as fugu and zebrafish [10]. The implications of such divergent N-terminal sequences on the functions of Runx proteins are not known.

The stability and activity of gnathostome Runx proteins are affected by several post-translational modifications including phosphorylation. In human RUNX1, the serine and threonine residues S249, S266, S276 and T273 followed by a proline residue act as phosphorylation sites for ERK [59], [60]. Among these sites, S249 and S276 are conserved in lamprey RunxB but not in the other two lamprey Runx proteins (Fig. 5). Phosphorylation at serine residue S104 within the Runt domain of human RUNX2 has been shown to negatively regulate RUNX2 activity by inhibiting its heterodimerization with RUNXβ [61]. This serine residue is invariably conserved among all gnathostome Runx. It is also conserved in all three lamprey Runx proteins. Additionally, the consensus phosphorylation site for CDC2, (S/T)PX(R/K), at which serine residue S451 of human RUNX2 was reported to be phosphorylated [62], is highly conserved in gnathostome Runx1–3 proteins. However, it is conserved only in lamprey RunxC and not in the other two lamprey Runx proteins (Fig. 5).

### Expression profile of Japanese lamprey α-subunit *Runx* genes

**Figure 6:**
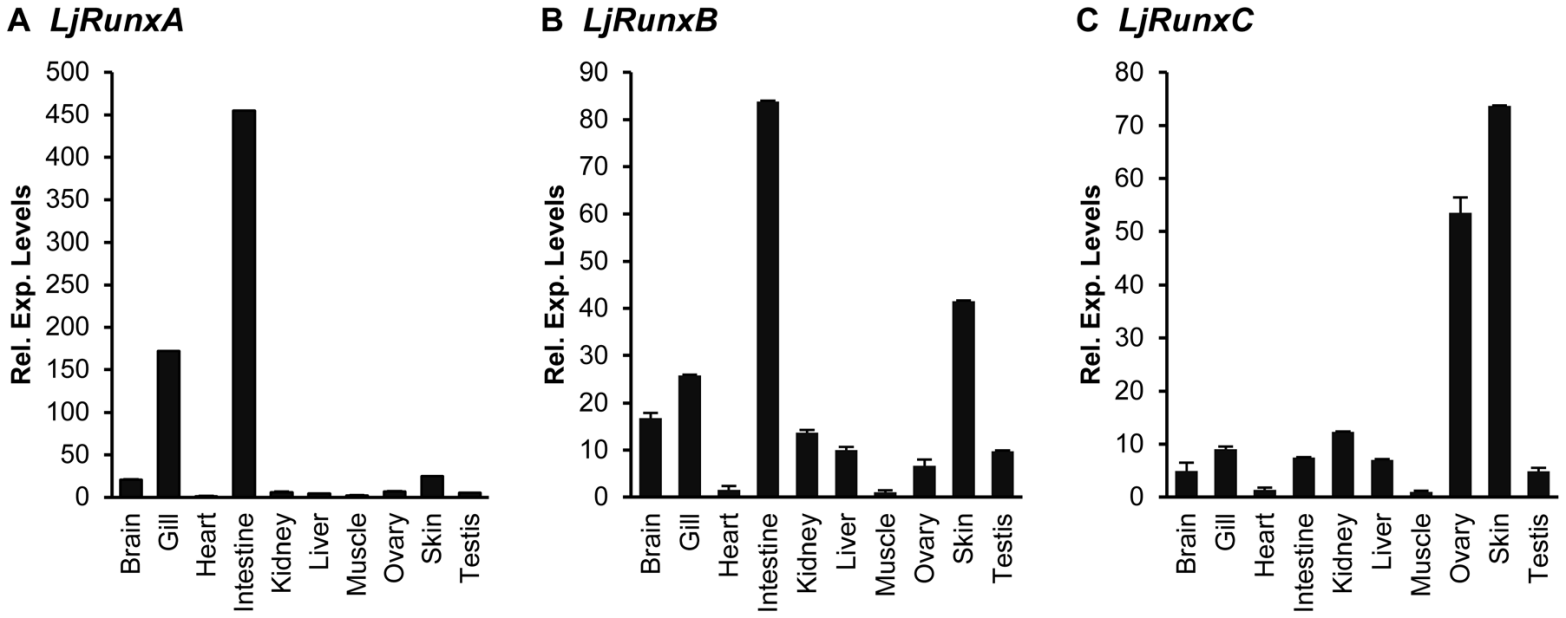
Expression patterns of Japanese lamprey α-subunit *Runx* genes. Relative expression levels of (A) *LjRunxA*, (B) *LjRunxB* and (C) *LjRunxC* in various tissues of the Japanese lamprey determined by qRT-PCR. Note that the relative expression levels of each *Runx* gene between different tissues are estimated in relation to the expression level of the tissue showing the lowest expression, and hence the expression levels are not comparable between different *Runx* genes.

We investigated the expression patterns of *Runx* genes in various tissues of adult Japanese lamprey by quantitative RT-PCR. *RunxA* and *RunxB* are highly expressed in the gills and intestine- tissues that comprise the primary lymphoid organs of the lamprey (Fig. 6A and B). In the lamprey, lymphocytes develop in the typhlosole, an invaginated spiral valve spanning the length of the intestine as well in the “thymoid”, the lamprey thymus-equivalent that is located at the tips of gill filaments [30]. *Runx* expression in the gills is reminiscent of that seen in the hagfish and amphioxus, where the gills and associated blood vessels are enriched with lymphocyte-like cells [4], [63]. Expression of *Runx* in the lymphoid compartments of these phylogenetically ancient chordates is consistent with its integral function in the specification of immune-related cells in gnathostomes, and may indicate a possible role for *Runx* in the primordial immune system of the chordate ancestor. Furthermore, significant *Runx* expression in the intestine of the adult lamprey appears to support the prevailing view of an ancestral function of *Runx* genes in the gut, since sea urchin [64], nematode (*C.elegans*) [65], amphioxus [4] and gnathostomes all express *Runx* in the developing gut.

Particularly striking is the significant expression of *RunxB* and *RunxC* in the skin of the lamprey (Fig. 6B and C). Apart from the primary lymphoid organs such as the gill and intestine, the lamprey epidermis is also home to abundant numbers of lymphocytes, in particular, the VLRC+ lymphocytes that display dendritic morphology and which have been posited to phenotypically resemble mammalian dendritic epidermal T cells (DETC) [31]. Though yet to be verified, based on the significant role *Runx3* plays in the development of DETC [66], it may be postulated that the lamprey *RunxC* similarly contributes to the specification of these immune cell types in the lamprey. In addition, the lamprey brain is another domain of significant *RunxB* expression (Fig. 6B). *Runx* expression in the brain and cranial ganglia has been previously demonstrated in the early larvae of the sea lamprey [33]. These expression patterns are consistent with those observed in the neuronal tissues of gnathostomes [27], [67], [68] as well as a basal metazoan, the sea anemone, *Nematostella* [11], suggestive of an evolutionarily ancient function in neural development.

Prominent expression of *RunxC* was also observed in the Japanese lamprey ovary (Fig. 6C). This expression pattern is concordant with *Runx* function in the female reproductive system of mammals [69], [70] and *Drosophila* [71] and may therefore hint at a possible role of *Runx* in conserved pathways of ovarian function. In gnathostomes, Runx proteins are central regulators in the development of cellular cartilage and ossification of endochondral bone [28], [72]. Expression of *Runx* has been described in the cartilage of vertebrates that lack mineralized bone, such as the hagfish and cartilaginous fishes [4], suggesting that *Runx* genes have a conserved functional role in cartilage formation in stem vertebrates. In the sea lamprey, Cattell *et. al* [33] detected *RunxA* and *RunxB* expression in the mucocartilage of larvae but noted the apparent absence of *Runx* expression in the branchial basket cartilage, skeletal elements that are believed to be homologous to the cellular cartilage of gnathostomes. This led to the speculation that *Runx* genes may have lost their ancestral function in cartilage development in the lamprey lineage or are not engaged in gene regulatory networks of the ancestral vertebrate cartilage [33]. Here, we have reported the presence of three *Runx* genes in the Japanese lamprey, as compared to only two previously identified in the sea lamprey. The characterization of the expression of all three *Runx* genes in the lamprey may shed new light into the involvement of *Runx* in early vertebrate skeletal development.

### Evolution of *Runx* family genes in vertebrates

Invertebrate chordates such as the amphioxus, *Branchiostoma floridae* (cephalochordate) and *Ciona intestinalis* (urochordate) contain a single *Runx* gene, known as *Runt* genes. The functions of these genes are not well characterized, although reports documenting *Runx* expression in the developing gut, pharyngeal endoderm and regenerating oral cirral skeletal cells of the amphioxus [4], [73] reflect functions in endodermal specification and roles in the rudimentary skeletogenic programs of these phylogenetically ancient chordates.

**Figure 7:**
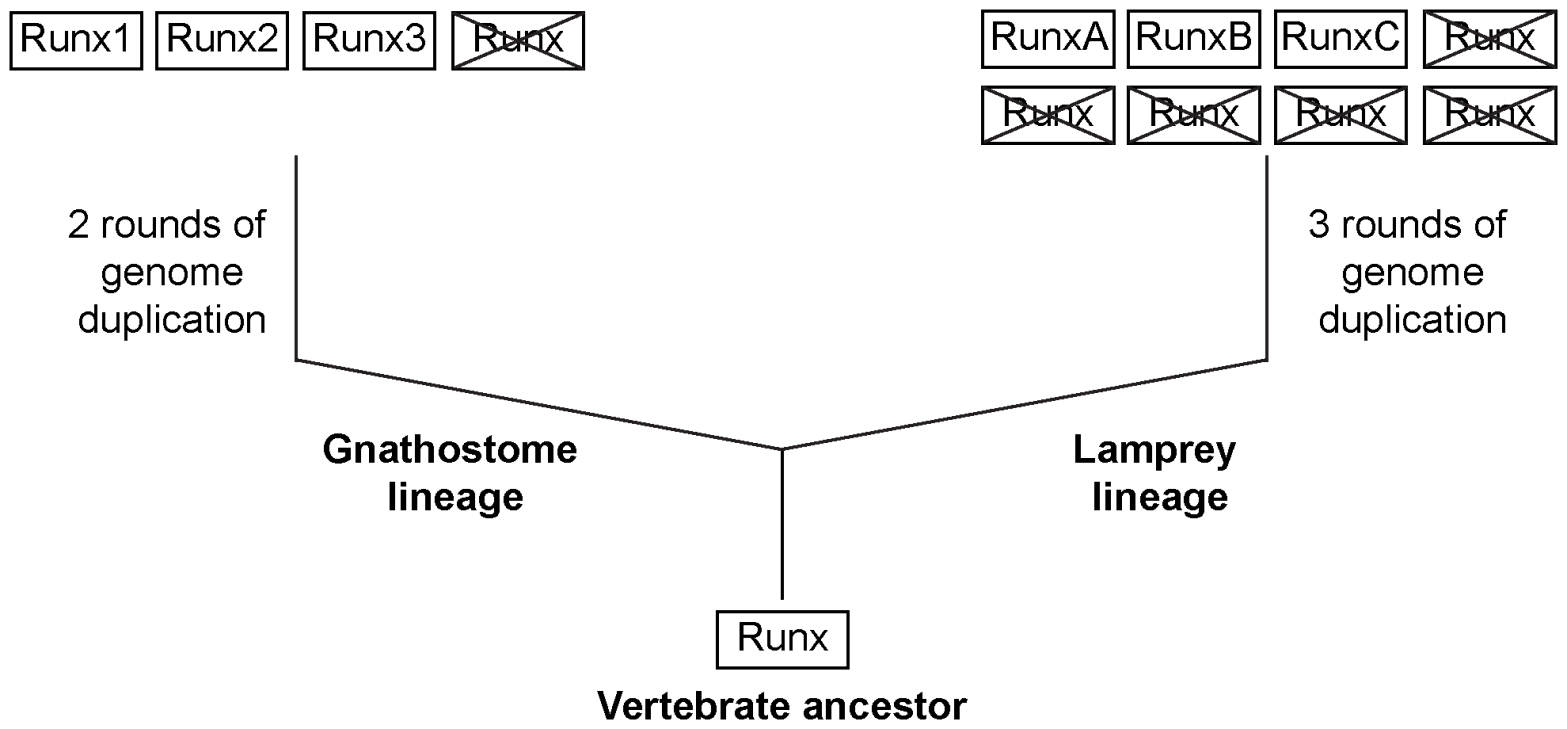
A model depicting the evolution of *Runx* genes in vertebrates. The phylogenetic analysis and synteny maps suggest that the three *Runx* genes in lamprey are not one-to-one orthologs of the three *Runx* genes in gnathostomes.

Based on the identification of the three *Runx* genes in the lamprey and their comparative analysis with *Runx* genes known in gnathostomes, we propose that the stem vertebrate ancestor contained a single *Runx* gene. As part of the two rounds of whole genome duplications, this gene locus underwent duplications (Fig. 7) in the lineage that gave rise to gnathostomes such as cartilaginous fishes, lobe-finned fishes and tetrapods giving rise to four *Runx* paralogs [6], [7]. Since these vertebrates contain only three *Runx* paralogs each (*Runx1*, *Runx2* and *Runx3*), we infer that the fourth paralog was lost in the common ancestor of gnathostomes. The remaining three *Runx* genes in the gnathostome ancestor acquired specialized and distinct functions such that *Runx1* is involved mainly in hematopoiesis [18], *Runx2* in skeletogenesis [19] and *Runx3* in neurodevelopment and immunity [24], [25], [27].

The whole-genome duplication history in the lamprey lineage is not well resolved. Although previous studies suggested that lampreys shared the two rounds of duplication with gnathostomes, a more recent study suggested that the two rounds of duplications may have occurred independently in the lamprey lineage followed by an additional round of whole genome duplication [35]. In any case, the presence of six *Hox* clusters in the Japanese lamprey and the relative age of paralogous genes in the lamprey genome as measured by the transversion rate of four-fold degenerate sites [35] suggests that its lineage has experienced three rounds of genome duplication. According to this hypothesis, the genome duplications would have given rise to eight paralogs of *Runx* genes in the lamprey lineage. Since only three *Runx* genes are present in the Japanese lamprey, we infer that five of the eight *Runx* genes have been lost (Fig. 7). Although the exact functions of the lamprey *Runx* genes have not been investigated, the differential expression patterns of *Runx* genes in various tissues of the Japanese lamprey (Fig. 6), sea lamprey [33] and the Atlantic hagfish [4] are suggestive of distinct functions of each of the cyclostome *Runx* genes. However, further investigations will be required to understand the specific functions of the *Runx* gene family in cyclostomes and how they differ from those in gnathostomes. Since lampreys lack mineralized tissues and an Ig-based adaptive immune system, some differences in the functions of gnathostome and lamprey *Runx* genes are expected.

### Characterization of a Japanese lamprey β-subunit *Runx* gene

**Figure 8:**
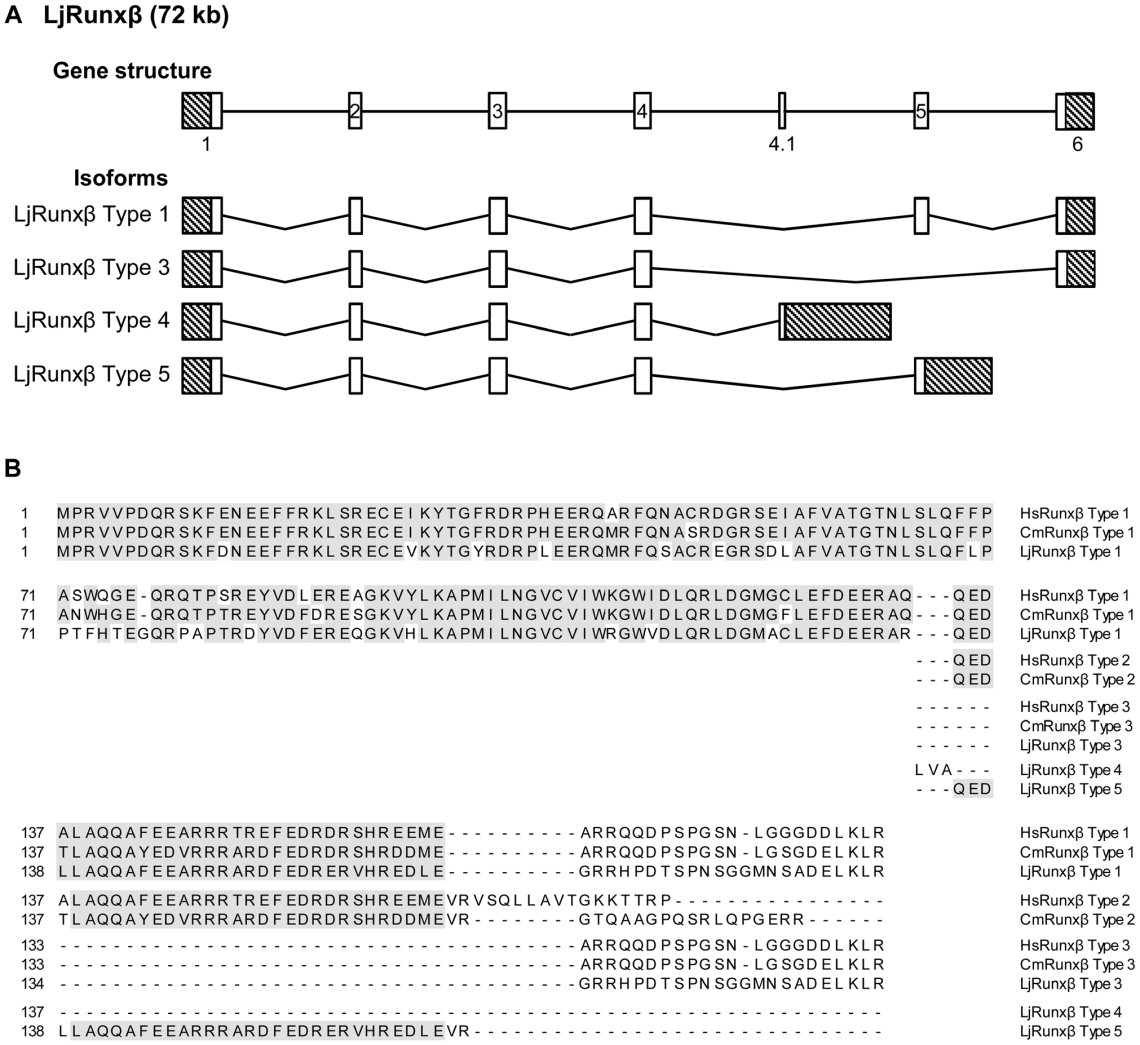
Exon-intron organization and protein sequence encoded by the Japanese lamprey *Runxβ*. (A) Schematic representation of the genomic structure and the four transcripts cloned (*LjRunxb* types 1, 3, 4 and 5). Exons are indicated by boxes. The 5′- and 3′-UTRs are represented as crosshatched boxes. (B) Alignment of Japanese lamprey, elephant shark and human RUNXβ amino acid sequences using ClustalW. Conserved residues are shaded grey. Hs, *Homo sapiens*; Cm, *Callorhinchus milii*; Lj, *Lethenteron japonicum*.

Gnathostome α-subunit Runx proteins form heterodimeric complexes with their β-subunit partner. This association not only enhances the DNA binding affinity of the complex by stabilizing the interaction of the α-subunit Runt domain with DNA, it also protects against the ubiquitin-mediated degradation of the α-subunit [74]. In addition to α-subunit *Runx* genes in the Japanese lamprey, we also cloned full-length coding sequence and various isoforms of the β-unit encoding *Runxb*. The genomic organization of the Japanese lamprey *Runxb* gene is largely similar to that of its gnathostome ortholog except for the presence of an additional exon 4.1 (Fig. 8A). Through alternative splicing, the gnathostome *Runxb* gene gives rise to three isoforms, *Runxb* Type 1, 2 and 3. Type 1 and Type 2 differ in several amino acids at their carboxyl termini while Type 1 and Type 3 isoforms are almost identical, except for the absence of exon 5 in Type 3 owing to exon skipping [1]. The Japanese lamprey *Runxb* is also transcribed into these three isoforms (Fig. 8A). In addition, we identified two isoforms (Type 4 and 5) that are unique to the Japanese lamprey (Fig. 8A). *Runxb* Type 4 terminates at the alternative exon 4.1, while *Runxb* Type 5 appears to be a truncated form of Type 1, terminating prematurely in exon 5 (Fig. 6A). The *Runxb* type 1, 3, 4 and 5 isoforms encode proteins of 189, 157, 137 and 168 amino acids, respectively (Fig. 6B). At the protein level, lamprey and gnathostome Runxβ show high conservation of amino acid residues 1–165 (Fig. 8B). Of these, the N-terminal 135 amino acids required for its heterodimerization with the α-subunit and stimulation of DNA-binding activity [2] as well as essential residues 68–93 for its interaction with filamin A in the cytoplasm [75] are almost perfectly conserved Functional significance of the various isoforms of gnathostomes Runxβ are not well documented, though one might speculate that Runxβ Type 3, which is missing the domain required for the heterodimerization with the α-subunit, may perform functions that are independent of its interaction with α-subunit Runx proteins. Such a function is supported by the recent observation that the retention of Runxβ in the cellular midbody during cytokinesis was not seen to be compromised in the absence of α-subunit Runx [76]. It remains to be demonstrated whether the various isoforms of lamprey Runxβ exhibit different functions.

## Supporting Information

Table S1 **Primers used for qRT-PCR of Japanese lamprey** ***Runx*** **genes.**
(PDF)

Figure S1 **Exon-intron organization of elephant shark and lamprey** ***Runx*** **genes.** Schematic representation of the gene structures of elephant shark *Runx1*, *Runx2* and *Runx3* and lamprey *RunxA*, *RunxB* and *RunxC*. Exons are indicated by boxes. Exons constituting the Runt domain are indicated in grey. The two alternative promoters are denoted as P1 and P2. Crosshatched boxes indicate 5′- and 3′-UTRs. The asterisk (*) indicates an exon in *LjRunxB* that is absent in mammals and different from exon 4.1 in elephant shark. Not drawn to scale.
(PDF)

Figure S2 **Phylogenetic analysis of chordate Runx sequences (Bayesian Inference).** Protein sequences of Japanese lamprey *Runx* genes were aligned with homologous sequences from selected chordates. A Bayesian inference (BI) tree was generated for the alignment. Statistical support values for the nodes are shown as Bayesian posterior probability values. Hagfish and Japanese lamprey Runx proteins are highlighted in red. Lancelet (*Branchiostoma floridae*) Runt (BfRunt) was used as the outgroup. Hs, *Homo sapiens*; Gg, *Gallus gallus*; Dr, *Danio rerio*; Cm, *Callorhinchus milii*; Sc, *Scyliorhinus canicula*; Mg, *Myxine glutinosa*; Lj, *Lethenteron japonicum*.
(PDF)

Figure S3 **Runx protein sequence alignment used for phylogenetic tree in** **Fig. 2** **and Fig. S2.** Alignment obtained after trimming the gaps using the Gblocks Server (ver. 0.91b). Hs, *Homo sapiens*; Gg, *Gallus gallus*; Dr, *Danio rerio*; Cm, *Callorhinchus milii*; Sc, *Scyliorhinus canicula*; Mg, *Myxine glutinosa*; Lj, *Lethenteron japonicum*; Bf, *Branchiostoma floridae*.
(PDF)

Figure S4 **Runx protein sequence alignment used for phylogenetic tree in Fig. S5.** Alignment obtained after trimming the gaps using the Gblocks Server (ver. 0.91b). Hs, *Homo sapiens*; Gg, *Gallus gallus*; Dr, *Danio rerio*; Cm, *Callorhinchus milii*; Sc, *Scyliorhinus canicula*; Mg, *Myxine glutinosa*; Lj, *Lethenteron japonicum*; Ci, *Ciona intestinalis*; Bf, *Branchiostoma floridae*; Nv, *Nematostella vectensis*.
(PDF)

Figure S5 **Phylogenetic analysis of chordate Runx sequences (including CiRunt and NvRunx).** Protein sequences of Japanese lamprey *Runx* genes were aligned with homologous sequences from selected chordates. A Maximum Likelihood (ML) tree employing the JTT+G model was generated for the alignment. Sea anemone (*Nematostella vectensis*) Runx (NvRunx) was used as the outgroup. Hs, *Homo sapiens*; Gg, *Gallus gallus*; Dr, *Danio rerio*; Cm, *Callorhinchus milii*; Sc, *Scyliorhinus canicula*; Mg, *Myxine glutinosa*; Lj, *Lethenteron japonicum*; Bf, *Branchiostoma floridae*; Ci, *Ciona intestinalis*.
(PDF)
