# Supplementary material for: Characterization of the Runx Gene Family in a Jawless Vertebrate, the Japanese Lamprey (Lethenteron japonicum)
Source: PLoS One. 2014 Nov 18;9(11):e113445. doi: 10.1371/journal.pone.0113445 (PMC4236176; doi:10.1371/journal.pone.0113445)
Supplement: Table S1 — Primers used for qRT-PCR of Japanese lamprey Runx genes. (PDF) [file pone.0113445.s001.pdf]

Table S1. Primers used for qRT-PCR of Japanese lamprey *Runx* genes. The actin gene was amplified as an internal control

Gene	Primer Name	Primer Sequence (5'→ 3')
LjRunxA	LjRunxA-FW	AATCGGAGGTGGAGCGCACGATCG
	LjRunxA-RV	GAGGTCGTTGAACCTCGCCACTTG
LjRunxB	LjRunxB-FW	CTCGCTGCTGCACTCGAAGCGCGCCA
	LjRunxB-RV	AGGTCGTTGAAGCGAGCCACCTGCTG
LjRunxC	LjRunxC-FW	AGCAGAGCAGCGAGATGCTGCGCAC
	LjRunxC-RV	CCACGTGACCCTTCATGACGGCAC
LjActin	LjActin-FW	ATCGTGCGTGACATCAAGGAGAAG
	LjActin-RV	GCGTACAGGTCCTTGCGGATGTC
