# Supplementary figures and images for: Characterization of the Runx Gene Family in a Jawless Vertebrate, the Japanese Lamprey (Lethenteron japonicum)
Source: PLoS One. 2014 Nov 18;9(11):e113445. doi: 10.1371/journal.pone.0113445 (PMC4236176; doi:10.1371/journal.pone.0113445)

## Elephant shark

**P1**

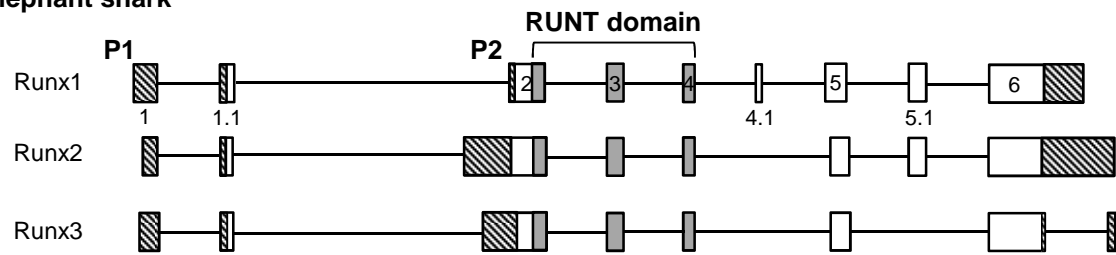

**Japanese lamprey**

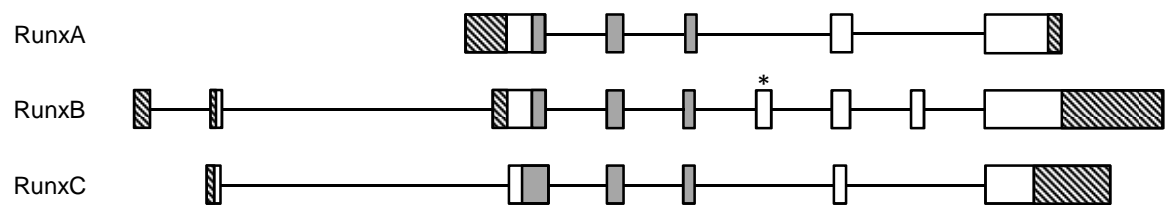

Supplement: Figure S1 — Exon-intron organization of elephant shark and lamprey Runx genes. Schematic representation of the gene structures of elephant shark Runx1, Runx2 and Runx3 and lamprey RunxA, RunxB and RunxC. Exons are indicated by boxes. Exons constituting the Runt domain are indicated in grey. The two alternative promoters are denoted as P1 and P2. Crosshatched boxes indicate 5′- and 3′-UTRs. The asterisk (*) indicates an exon in LjRunxB that is absent in mammals and different from exon 4.1 in elephant shark. Not drawn to scale. (PDF) [file pone.0113445.s002.pdf]

**Figure S2**

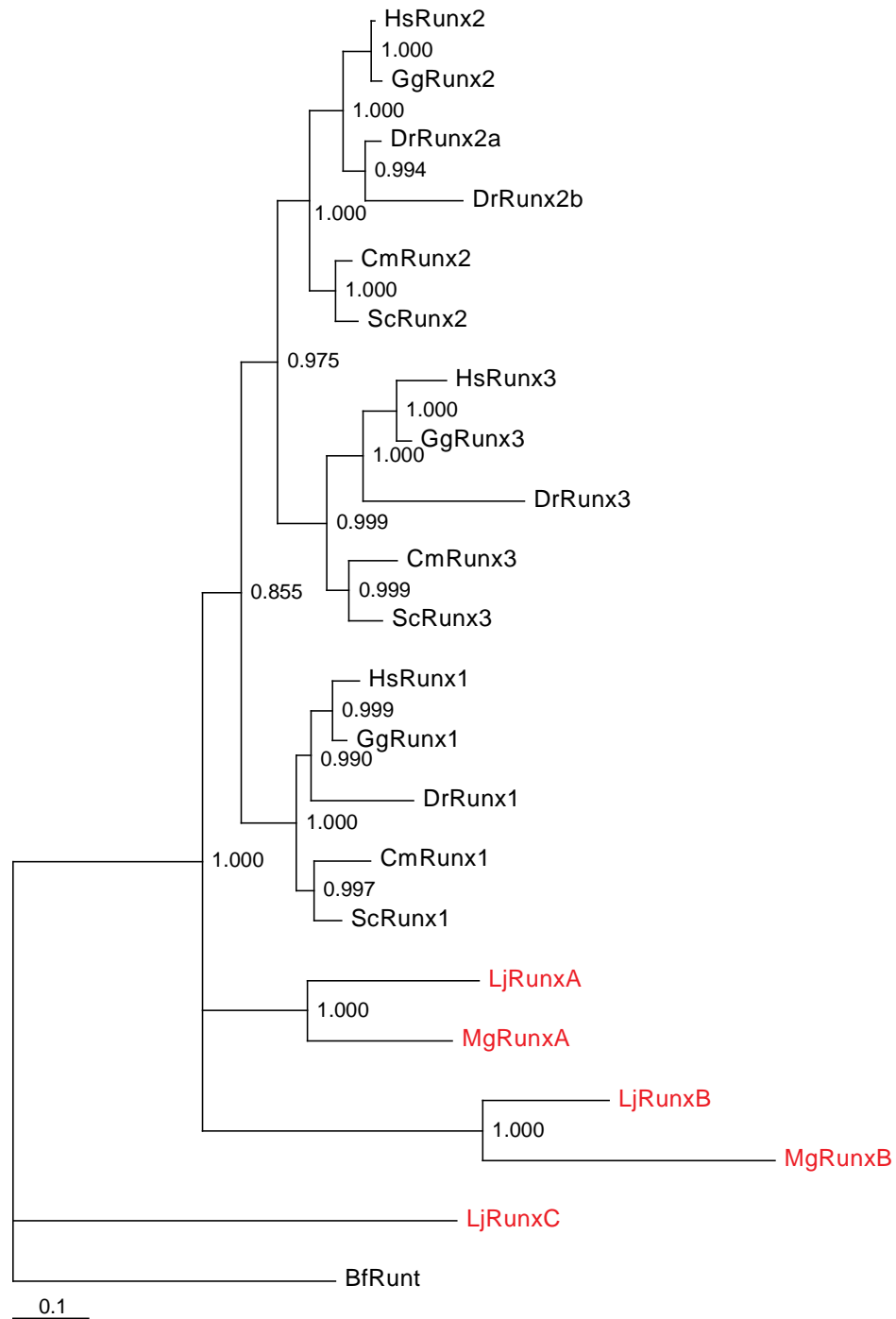

Supplement: Figure S2 — Phylogenetic analysis of chordate Runx sequences (Bayesian Inference). Protein sequences of Japanese lamprey Runx genes were aligned with homologous sequences from selected chordates. A Bayesian inference (BI) tree was generated for the alignment. Statistical support values for the nodes are shown as Bayesian posterior probability values. Hagfish and Japanese lamprey Runx proteins are highlighted in red. Lancelet (Branchiostoma floridae) Runt (BfRunt) was used as the outgroup. Hs, Homo sapiens; Gg, Gallus gallus; Dr, Danio rerio; Cm, Callorhinchus milii; Sc, Scyliorhinus canicula; Mg, Myxine glutinosa; Lj, Lethenteron japonicum. (PDF) [file pone.0113445.s003.pdf]

**Figure S5**

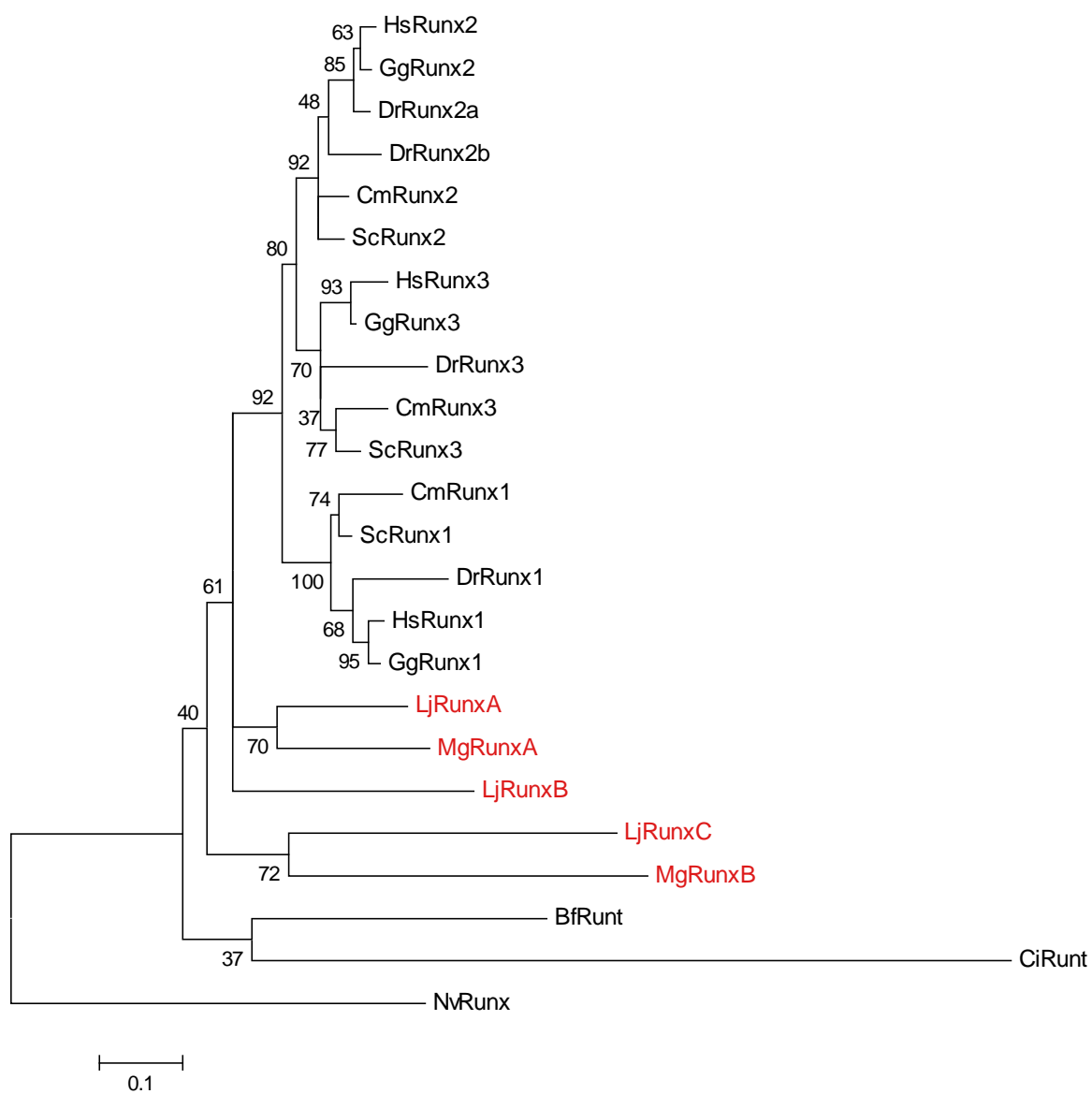

Supplement: Figure S5 — Phylogenetic analysis of chordate Runx sequences (including CiRunt and NvRunx). Protein sequences of Japanese lamprey Runx genes were aligned with homologous sequences from selected chordates. A Maximum Likelihood (ML) tree employing the JTT+G model was generated for the alignment. Sea anemone (Nematostella vectensis) Runx (NvRunx) was used as the outgroup. Hs, Homo sapiens; Gg, Gallus gallus; Dr, Danio rerio; Cm, Callorhinchus milii; Sc, Scyliorhinus canicula; Mg, Myxine glutinosa; Lj, Lethenteron japonicum; Bf, Branchiostoma floridae; Ci, Ciona intestinalis. (PDF) [file pone.0113445.s006.pdf]
