# Supplementary material for: Characterization of the Runx Gene Family in a Jawless Vertebrate, the Japanese Lamprey (Lethenteron japonicum)
Source: PLoS One. 2014 Nov 18;9(11):e113445. doi: 10.1371/journal.pone.0113445 (PMC4236176; doi:10.1371/journal.pone.0113445)
Supplement: Figure S3 — Runx protein sequence alignment used for phylogenetic tree in Fig. 2 and Fig. S2. Alignment obtained after trimming the gaps using the Gblocks Server (ver. 0.91b). Hs, Homo sapiens; Gg, Gallus gallus; Dr, Danio rerio; Cm, Callorhinchus milii; Sc, Scyliorhinus canicula; Mg, Myxine glutinosa; Lj, Lethenteron japonicum; Bf, Branchiostoma floridae. (PDF) [file pone.0113445.s004.pdf]

Figure S3

```
1    DASTSRRFTPPSTALSPGLR--SGDRSMVEVLADHPGELVRTDSPNFLCSVLPTHWRCNKTLPIAFKVVALGDVPDGTLVTVMAGNDENY   HsRUNX1
1    DTSTSRRFTPPSTALSPGLR--AADRSMVEVLADHPGELVRTDSPNFLCSVLPTHWRCNKTLPIAFKVVALGDVPDGTLVTVMAGNDENY   GgRunx1
1    EPAPGRRFTPPSTTLSSGLR--MADRSMVEVLSDHPGELVRTDSPNFLCSVLPTHWRCNKTLPIAFKVVALGDIPDGTLVTVMAGNDENY   DrRunx1
1    DPSTSRRFPPPCTSLGSGLR--PADRTMVDVLADHPGELVRTDSPNFLCSVLPTHWRCNKTLPIAYKVVSLGDVPDGTLVTVLAGNDENY   CmRunx1
1    DTNTSRRFTPPSTTLSTGLR--ATDRSMVDVLADHPGELVRTDSPNFLCSVLPTHWRCNKTLPIAFKVVALGDVPDGTLVTVLAGNDENY   ScRunx1
1    DPSTSRRFSPPSSSLQPGLRPPHDNRTMVEIIADHPAELVRTDSPNFLCSVLPSHWRCNKTLPVAFKVVALGEVPDGTVVTVMAGNDENY   HsRUNX2
1    DPSTSRRFSPPSSSLQPGLR-PHDNRTMVEIIADHPAELVRTDSPNFLCSVLPSHWRCNKTLPVAFKVVALGEVPDGTVVTVMAGNDENY   GgRunx2
1    DPSTSRRFSPPSSSLQP-LR-PHDNRTMVEIIADHPAELVRTDSPNFLCSVLPSHWRCNKTLPVAFKVVALGDVPDGTVVTVMAGNDENY   DrRunx2a
1    DPSAGRRFSPPVSVKMN-LR-AQENRSMAEIIADHPAELVRTDSPNFLCSVLPSHWRCNKTLPVAFKVVALGEVPDGTVVTVMAGNDENY   DrRunx2b
1    DPSTSRRFTPPSTTLPASLIRPHENRSMVDIIADHPAELVRTDSPNFLCSVLPSHWRCNKTLPVAFKVVALGDVPDGTVVTVMAGNDENY   CmRunx2
1    DPTTSRRFTPPSTTLPASLIRPHENRTMVDIIADHPAELVRTDSPNFLCSVLPSHWRCNKTLPVAFKVVALGDVPDGTVVTVMAGNDENY   ScRunx2
1    DPSTSRRFTPPSPAFPCGGRARPEVRSMVDVLADHAGELVRTDSPNFLCSVLPSHWRCNKTLPVAFKVVALGDVPDGTVVTVMAGNDENY   HsRUNX3
1    DPSTSRRFTPPSTAFPCGGRARPEVRSVVDVLADHAGELVRTDSPNFLCSVLPSHWRCNKTLPVAFKVVALGDVPDGTVVTVMAGNDENY   GgRunx3
1    DPSTSRRFTPPSASFPCAGRP-VESRSVVDVLADHAGELVRTDSPNFLCSVLPSHWRCNKTLPVAFKVVALGDVPDGTLVTVMAGNDENY   DrRunx3
1    DPNTSRRFTPPSTTLSSS-RVRPETRTVVDVLADHAGELVRTDSPNFLCSVLPSHWRCNKTLPVAFKVVALGDVPDGTLVTVMAGNDENY   CmRunx3
1    DPSTSRRFTPPSTTLTPS-RIRPETRTVVDVLQDHAGELVRTDSPNFLCSVLPSHWRCNKTLPVAFKVVALGDVPDGTLVTVMAGNDENY   ScRunx3
1    DAGVSRRFTPPSTAL---IGGGAHDRPMGDVLADHPGELVRTDSPNFLCSVLPSHWRCNKTLPVAFKVVALGDVPDGTLVGVMAGNDENY   LjRunxA
1    DTGHTRRFTPPSNTLTPNLRPG--DRPMVEVLADHPGELVRTDSPNFLCSVLPSHWRCNKTLPVAFRVVALGDVADGTMVTVMAGNDENY   MgRunxA
1    HHHHHHLQQQQQQLGTGGSLLHSKRATADVLADHPGELVRTDSPNFLCSVLPSHWRCNKTLPVAFKVVSLGGVPDGTVVTVLAGNDENY   LjRunxB
1    -RHHHSTQGGSVTTPGPG-----VECVPIDILAEHAAELVRTDSPGILCSVLPSHWRCNKTLPVPFKVLCLVDVPDGTEVGVLAGNDENC   MgRunxB
1    QQQPSAPCSPPLSLGKLQGGAPPEEAGLALGLGEQSSEMLRTDSPNFLCSALPPHWRCNKTLPVAFKVMAMADVPDGTPVAVMAGNDENY   LjRunxC
1    ---DSRRFSPFADPGKMG------DRGLVDALADHPGELVRTDSPNFVCSVLPSHWRCNKTLPVPFKVVALGDIPDGTLVTVMAGNDENY   BfRunt

89   SAELRNATAAMKNQVARFNDLRFVGRSGRGKSFTLTITVFTNPPQVATYHRAIKITVDGPREPRRHRQKLDDKPGSLSFSERLSELEQLR   HsRUNX1
89   SAELRNATAAMKNQVARFNDLRFVGRSGRGKSFTLTITVFTNPPQVATYHRAIKITVDGPREPRRHRQKLDEKPGSLSFSERLSELEQLR   GgRunx1
89   SAELRNATAAIKNQVARFNDLRFVGRSGRGKSFTLTITVFTNPPQVATYQRAIKITVDGPREPRRHRQKPDEKPGALAFSE------QLR   DrRunx1
89   SAELRNATAVMKNQVARFNDLRFVGRSGRGKSFTLTITVFTNPPQVATYHRAIKITVDGPREPRRHRQKLEEKSAGMAFSERLSELEQIR   CmRunx1
89   SAELRNATAVMKNQVARFNDLRFVGRSGRGKSFTLTITVFTNPPQVATYHRAIKITVDGPREPRIHRQKLEEKNASLAFSERLSELEQLR   ScRunx1
91   SAELRNASAVMKNQVARFNDLRFVGRSGRGKSFTLTITVFTNPPQVATYHRAIKVTVDGPREPRRHRQKLDDKPS-L-FSDRLSDLGRIP   HsRUNX2
90   SAELRNASAVMKNQVARFNDLRFVGRSGRGKSFTLTITVLTNPPQVATYHRAIKVTVDGPREPRRHRQKLDDKPS-L-FPERLSDLGRIP   GgRunx2
89   SAELRNASAVMKNQVARFNDLRFVGRSGRGKSFTLTITVFTNPPQVATYHRAIKVTVDGPREPRRHRQKLEDKPP-L-FSERLSELERLR   DrRunx2a
89   SAELRNASGVMKNQVARFNDLRFVGRSGRGKSFTLTITVFTNPPQVATYHRAIKVTVDGPREPRRHRPKLDDKAG-L-FSDRLSELERIR   DrRunx2b
91   SAELRNASAVMKNQVARFNDLRFVGRSGRGKSFTLTITVFTNPPQVATYHRAIKVTVDGPREPRRHRQKPEDKVG-L-FSERLSELERLR   CmRunx2
91   SAELRNASAVMKNQVARFNDLRFVGRSGRGKSFTLTITVFTNPPQVATYHRAIKVTVDGPREPRRHRQKPEDKVG-L-FSERLSELERLR   ScRunx2
91   SAELRNASAVMKNQVARFNDLRFVGRSGRGKSFTLTITVFTNPTQVATYHRAIKVTVDGPREPRRHRQKLEDKP----FPDRFGDLERLR   HsRUNX3
91   SAELRNASAVMKNQVARFNDLRFVGRSGRGKSFTLTITVFTNPTQVATYHRAIKVTVDGPREPRRHRQKLEDKP----FADRYGELERLR   GgRunx3
90   SAELRNASAVMKNQVARFNDLRFVGRSGRGKSFTLTITVFTGPPQVATYHRAIKVTVDGPREPRRHRVKPDDKQ----FSDRLSDIERFQ   DrRunx3
90   SAELRNASAVMKNAVARFNDLRFVGRSGRGKSFTLTITVFTSPPQVATYHRAIKVTVDGPREPRRHRQKLEDKPNTL-FSDRLSELERYR   CmRunx3
90   SAELRNASAVMKNTVARFNDLRFVGRSGRGKSFTLTITVFTSPPQVATYHRAIKVTVDGPREPRRHRQKLEDKPGTL-FSDRLSELERYR   ScRunx3
88   SAELRNASAVMKNQVARFNDLRFVGRSGRGKSFTLTITVFTSPPQVATYHRAIKVTVDGPREPRRHRQRLEDKAGAAMFPERLCELEQFR   LjRunxA
89   SAELRNASAVIKNQVARFNDLRFVGRSGRGKSFTLTITVFSNPPQVATYHRAIKVTVDGPREPRRHRQRMEEKAGGSLFPDRLCDLDQLR   MgRunxA
91   AAELRNATAVMKQQVARFNDLRFVGRSGRGKSFTLTITVFTSPPQVATYHRAIKVTVDGPREPRIHRQRLED--KALSFSERLSELEHLR   LjRunxB
85   TAELRNTRAVTKERVARFNDLRFVGRSGRGKSLTLTITVFTRPPQVATYQRAIKVTVDGPREPRRHRQRPEE---PLPFTD----LEHYC   MgRunxB
91   SAELRNASAVMKGHVARFNDLRFVGRSGRGKSLNLTITVFTSPPQVATYQRAIKVTVDGPREPRRHRQRQED-LKSNSMGGLFPER-LLQ   LjRunxC
82   SAELRNNQAVMKNQVARFNDLRFVGRSGRGKSFTLTITVFTSPPQVATYHRAIKVTVDGPREPRRHRQKLEE-KHALSFSERLSELGLER   BfRunt

179  RTAMRVSPSTAFNPQPQSQMPPWSYDQSYPDLTAFFPALPSISD-----PRMHYPGAFTYSPTPVTSGSAMGS-ATRYHTYLPPPYPQGG   HsRUNX1
179  RTAMRVSPTTAFNPQPQSQIPPWSYDQSYSDLTAFFSALPSISD-----PRMHYPGAFTYTPTPVSSGSAMST-ATRYHTYLPPPYPQGS   GgRunx1
173  RSAMRCSP-PPFGSPAHSQIPSWSYEQSYPELTAFFPSLPSLPDGRFSDPRVPYPGAFTYTPTPVTSASAMSSPAGRYHTYLPPAYPQAG   DrRunx1
179  RSAMRGSPTTAFNPQAHSQIPPWTYDQSYSDLTAFFSALPPLSDSRFPDPRMHYPGAFAYTPTPVTNGSAMTT-AARYHTYLPPPYPQGG   CmRunx1
179  RSAMRLSPTTAFNPQAQSQIPPWTYDQSYSDLTAFFSALPSLSDGRFTDPRMHYPGPFPYTPTPVTTGSAMTT-AARYHTYLPPPYPQTG   ScRunx1
179  HPSMRVGVPSPFNPQGQSQIPPWSYDQSYSELGPFFPSISSLTESRFSNPRMHYPATFTYTP-PVTSGSLGMSATTHYHTYLPPPYPQSG   HsRUNX2
178  HPSMRVGVPSPFNPQGQSQIPPWSYDQSYSELGPFFTSISSLTESRFSNPRMHYPATFTYTP-PVTSGSLGMSATTHYHTYLPPPYPQSG   GgRunx2
177  QTTMRVAVPNSFNPQGQTQIPPWSYEQPYSELSPFFPSLSSLTDSRFPSPRMHYPATFTYTPTPVTSGSLGMSTTTHYHTYLPPPYPQSG   DrRunx2a
177  QTTMRVTVPNSYTPQGQTQIPPWSYDQTYTDLSPFFPAFSSLTESRFSSPRMHYPATFTYTPTPVTTGSLG---SAHYHTYLPPPYPQSG   DrRunx2b
179  QTAMRVGAPVPFSSQGQAQIPPWSYDQSYSDLGAFQFGLSSLTDSRFSNPRMHYPATFTYTPTPVTSGSLGMSATTHYHTYLPPPYPQSG   CmRunx2
179  QTAMRVGAPAPFSPQATSQI---------TDF-HFQFGLSSLTESRFPNPRMHYPAAFTYTPTPVTTGSLGMSAATHYHTYLPPPYPQSG   ScRunx2
176  ---MRVTPTSHFSSQPQTPI---------SDPRQFFPTLPTLTESRFPDPRMHYPAAFPYSATPSGTSVAGMPATSRFHTYLPPPYPQSG   HsRUNX3
177  QS-MRVTPPSHFGSQAQTPI---------SDPRQFFPTLPTLTETRFSDPRMHYPATFPYTATPSATGMTSMPTTARFHTYLPPPYPQSG   GgRunx3
176  RASLRMNPPGLWPDQIDPPTLRW------TDLFQQFPSLSPLTAPRFSDSHMHYPSHFTYSANPSSTGVAGMPTSSRYHTYLPPPYPQNS   DrRunx3
179  QTAMRVGPPSHFSSSAQTQM---------SDPRQFFPGLSSLADSRFADPRVHYPAAFTYSATPPTTGMSSMASTARYHTYLPPPYPQGG   CmRunx3
179  QTTMRVGPPTHFSPQAQTQM---------ADHRQFFPGLSSLTESRFSDPRMNYPAAFTYATTPSTGGMTGMAAAARYHTYLPPPYPQSG   ScRunx3
178  RSGMRVPPHTAFNAQPQPQM---------SELGAFAAGLSSLPEGRFGDPRVHYPAYTPGGAASSALGVAAAAAAARYHHHLPPPYPQGS   LjRunxA
179  RPTVRLSPPGAFNAQPQIQL---------GELGALAAGLSSLSDSRFSDPRMHYPFHYPTAAPTSAAPAVATAAAARYHYHLPPPCPQVP   MgRunxA
179  RTALRVGARPPLNP--PPHYPPWSFDQPYSELGPFFASLSYLPDSRNTDPRMHYSARYAYLPPPYPAHSPFNAASAPFTHFSAGPAPSTA   LjRunxB
168  RSSIRHG-QTPLNP--SSHY---------TEPGTCLSAIPSLPDSHYGDSRLHCP-RYTYLPPPYPTNASFSSTSTPFQHFSASPTPCSA   MgRunxB
179  LG------QGVFNP---------------HHDGQF-------------DPRLHYP--FAYP---------APPPSPRYHAYLPPPYP---   LjRunxC
171  LRHSHIAQPSAYTPNPQIQAPSSWPYQPYPADSQF-----RFSDPRLSDPRLLYPGFTAYSSGPTTTM--TSLTSPRYLPMSPPGFPQLG   BfRunt

263  PFQASSPSYHLYYGASGSYQFSMV--RSPPRILPPCTNASGSALLNPSLPNQSD-VEAEGSHSNSPTARLEEAVWRPY   HsRUNX1
263  PFQTSSPSYHLYYGTSGSYQFSMIS-RSPPRIHPPCTNASGSTLLNPNLPNQSD-VEAEGSHSNSPTARLEEAVWRPY   GgRunx1
262  AFQASSSPYHLYYSSAGSYQFSMMPSRSPPRILP-CTNASGSALLHPSLPNQSEGVEAEGSHSSSPT---VEAVWRPY   DrRunx1
268  HFQTSSAPYHLYYGTSGSYQFSMMS-RSPPRILPPCTNASGSSLLNPSLPNQNE-GETNGSHSNSPTARLEEGVWRPY   CmRunx1
268  PFQTSSSPYHLYYGTSGSYQFSMMT-RSPPRILPPCTNASGSSLLNPSLPNQSD-VDADGSHSNSPTGRLEEAVWRPY   ScRunx1
268  PFQTSSTPY-LYYGTSGSYQFPMVPGRSPSRMLPPCTTTSGSTLLNPNLPNQNDGVDADGSHSSSPTGRMDESVWRPY   HsRUNX2
267  PFQTSSTPY-LYYGTSGSYQFPMVPGRSPSRMLPPCTTTSGSTLLNPNLPNQSDGVEADGSHSSSPTGRMDESVWRPY   GgRunx2
267  PFQTSSTPY-LYYGASGSYQFPMVPGRSPSRMLPPCTSASGSTLINPNLPNQTDGGEADGSHSSSPTGRMDESVWRPY   DrRunx2a
264  PFQSSSTPY-LYYGASGSYQFSMVPGRSPTRMMPPCTSASGTSVVNPNLPVQADGVEGDGSHSNSPTGRMDEGVWRPY   DrRunx2b
269  HFQTSTAPY-LYYGTSGSYQFSMVAGRSPSRMLP-CTSASGSTLLNPNLPNQNDGVDADGSHSSSPTGRIDESVWRPY   CmRunx2
259  PFQTSSAPY-LYYGTSGSYQFSMVAGRSPSRMLPPCTSASGSTLLNPNLPNQNDGVDADGSHSNSPTGRIDESVWRPY   ScRunx2
255  PFQANPSPYHLYYGTSGSYQFSMVAGRSPTRMLASCTSS-AGNLMNPSLGGQSDGVEADGSHSNSPTGRMDEAVWRPY   HsRUNX3
257  PFQTNPSPYHLYYGTSGSYQFSMVAGRSPTRMLSSCTS--GNNLMNPNLGNQNDGVDADGSHSNSPTGRMDESVWRPY   GgRunx3
260  HFQTNSSPYHLYYGTGGSYQFSMIPTRSPTRMLTSCTAAGNNNLINANLGNQSDGVDADGSHSNSPTTRIDESVWRPY   DrRunx3
260  TFQAGASPYHLYYGTSGSYQFSMVAGRSPTRMLPSCTS--GSSLMNPNLANQNDGVDPNGSQSNSPTGRLDESVWRPY   CmRunx3
260  PFQTSVSPYHLYYGASGSYQFSMVAGRSPTRMLPSCTS--GSTLMNPNLANQNDVVDADGSQSNSPTGRMDESVWRPY   ScRunx3
259  AFQAAASAQHLYYGAASSYQFPPPVMQQPGAVGEERPLATAPLINNPALPSAHQDHDPEGSQGSSPTGRLEEAVWRPY   LjRunxA
260  PFQSTSTPYHLYYGASGSYQFPMVAGRSPGRG---------------VLLSGLPGTDAGTGHRSSPPSRLDETVWRPY   MgRunxA
267  PFQSSSAS-QLYYGHSPAYSLSVMAGRQSAVGGRMVATG-DSVLLTPGLSSPDGDDGGDGSSSSSPSRLEPESVWRPY   LjRunxB
245  PFGSGSSSYPLYYSHSASYQLSIIEGRPPVHHHQTFPTMGSSVLLTPGLSSSDGDVEGDGSNENSPSRLDHETVWRPY   MgRunxB
221  AYQNGGPPYALFYG--------APPSYQLSLVAGASDQRS--PELPGSVGACAEGTESDGSCPSVSPRAMEEAVWRPY   LjRunxC
254  NYSPGSPPYHHLYGGS--YQYPILPGREPSILAATSNSQT-KPQLLPAQEKEASSQPGNGNHGNRQQPEKRETVWRPY   BfRunt
```
