# Supplementary material for: Characterization of the Runx Gene Family in a Jawless Vertebrate, the Japanese Lamprey (Lethenteron japonicum)
Source: PLoS One. 2014 Nov 18;9(11):e113445. doi: 10.1371/journal.pone.0113445 (PMC4236176; doi:10.1371/journal.pone.0113445)
Supplement: Figure S4 — Runx protein sequence alignment used for phylogenetic tree in Fig. S5. Alignment obtained after trimming the gaps using the Gblocks Server (ver. 0.91b). Hs, Homo sapiens; Gg, Gallus gallus; Dr, Danio rerio; Cm, Callorhinchus milii; Sc, Scyliorhinus canicula; Mg, Myxine glutinosa; Lj, Lethenteron japonicum; Ci, Ciona intestinalis; Bf, Branchiostoma floridae; Nv, Nematostella vectensis. (PDF) [file pone.0113445.s005.pdf]

Figure S4

```
1    DASTSRRFPSTALRSMVEVLADHPGELVRTDSPNFLCSVLPTHWRCNKTLPIAFKVVALGDVPDGTLVTVMAGNDENYSA   HsRUNX1
1    DTSTSRRFPSTALRSMVEVLADHPGELVRTDSPNFLCSVLPTHWRCNKTLPIAFKVVALGDVPDGTLVTVMAGNDENYSA   GgRunx1
1    EPAPGRRFPSTTLRSMVEVLSDHPGELVRTDSPNFLCSVLPTHWRCNKTLPIAFKVVALGDIPDGTLVTVMAGNDENYSA   DrRunx1
1    DPSTSRRFPCTSLRTMVDVLADHPGELVRTDSPNFLCSVLPTHWRCNKTLPIAYKVVSLGDVPDGTLVTVLAGNDENYSA   CmRunx1
1    DTNTSRRFPSTTLRSMVDVLADHPGELVRTDSPNFLCSVLPTHWRCNKTLPIAFKVVALGDVPDGTLVTVLAGNDENYSA   ScRunx1
1    DPSTSRRFPSSSLRTMVEIIADHPAELVRTDSPNFLCSVLPSHWRCNKTLPVAFKVVALGEVPDGTVVTVMAGNDENYSA   HsRUNX2
1    DPSTSRRFPSSSLRTMVEIIADHPAELVRTDSPNFLCSVLPSHWRCNKTLPVAFKVVALGEVPDGTVVTVMAGNDENYSA   GgRunx2
1    DPSTSRRFPSSSLRTMVEIIADHPAELVRTDSPNFLCSVLPSHWRCNKTLPVAFKVVALGDVPDGTVVTVMAGNDENYSA   DrRunx2a
1    DPSAGRRFPVS--RSMAEIIADHPAELVRTDSPNFLCSVLPSHWRCNKTLPVAFKVVALGEVPDGTVVTVMAGNDENYSA   DrRunx2b
1    DPSTSRRFPSTTLRSMVDIIADHPAELVRTDSPNFLCSVLPSHWRCNKTLPVAFKVVALGDVPDGTVVTVMAGNDENYSA   CmRunx2
1    DPTTSRRFPSTTLRTMVDIIADHPAELVRTDSPNFLCSVLPSHWRCNKTLPVAFKVVALGDVPDGTVVTVMAGNDENYSA   ScRunx2
1    DPSTSRRFPSPAFRSMVDVLADHAGELVRTDSPNFLCSVLPSHWRCNKTLPVAFKVVALGDVPDGTVVTVMAGNDENYSA   HsRUNX3
1    DPSTSRRFPSTAFRSVVDVLADHAGELVRTDSPNFLCSVLPSHWRCNKTLPVAFKVVALGDVPDGTVVTVMAGNDENYSA   GgRunx3
1    DPSTSRRFPSASFRSVVDVLADHAGELVRTDSPNFLCSVLPSHWRCNKTLPVAFKVVALGDVPDGTLVTVMAGNDENYSA   DrRunx3
1    DPNTSRRFPSTTLRTVVDVLADHAGELVRTDSPNFLCSVLPSHWRCNKTLPVAFKVVALGDVPDGTLVTVMAGNDENYSA   CmRunx3
1    DPSTSRRFPSTTLRTVVDVLQDHAGELVRTDSPNFLCSVLPSHWRCNKTLPVAFKVVALGDVPDGTLVTVMAGNDENYSA   ScRunx3
1    DAGVSRRFPSTALRPMGDVLADHPGELVRTDSPNFLCSVLPSHWRCNKTLPVAFKVVALGDVPDGTLVGVMAGNDENYSA   LjRunxA
1    DTGHTRRFPSNTLRPMVEVLADHPGELVRTDSPNFLCSVLPSHWRCNKTLPVAFRVVALGDVADGTMVTVMAGNDENYSA   MgRunxA
1    GGGAGRRFPAAAKRATADVLADHPGELVRTDSPNFLCSVLPSHWRCNKTLPVAFKVVSLGGVPDGTVVTVLAGNDENYAA   LjRunxB
1    DTPGRHHHGGSVTCVPIDILAEHAAELVRTDSPGILCSVLPSHWRCNKTLPVPFKVLCLVDVPDGTEVGVLAGNDENCTA   MgRunxB
1    DHHHHQQQPSAPCAGLALGLGEQSSEMLRTDSPNFLCSALPPHWRCNKTLPVAFKVMAMADVPDGTPVAVMAGNDENYSA   LjRunxC
1    DPYSNLPYPNPCLRHVVDILSDHQGELIKTDSPNFLCTPLPQHWRVNKSLQTPFKVVALSDIPDGTTVTVMAGNDENYSA   CiRunt
1    ---DSRRFFA---RGLVDALADHPGELVRTDSPNFVCSVLPSHWRCNKTLPVPFKVVALGDIPDGTLVTVMAGNDENYSA   BfRunt
1    -------------RSLVEALAEYPGELVKTDSPNFVCSVLPSHWRCNKTLPVAFKVVSLGDIPDGVIVSIAAGNDENFVA   NvRunx

81   ELRNATAAMKNQVARFNDLRFVGRSGRGKSFTLTITVFTNPPQVATYHRAIKITVDGPREPRRFSERLSELEQLRRTAMR   HsRUNX1
81   ELRNATAAMKNQVARFNDLRFVGRSGRGKSFTLTITVFTNPPQVATYHRAIKITVDGPREPRRFSERLSELEQLRRTAMR   GgRunx1
81   ELRNATAAIKNQVARFNDLRFVGRSGRGKSFTLTITVFTNPPQVATYQRAIKITVDGPREPRRFSEQL------RRSAMR   DrRunx1
81   ELRNATAVMKNQVARFNDLRFVGRSGRGKSFTLTITVFTNPPQVATYHRAIKITVDGPREPRRFSERLSELEQIRRSAMR   CmRunx1
81   ELRNATAVMKNQVARFNDLRFVGRSGRGKSFTLTITVFTNPPQVATYHRAIKITVDGPREPRIFSERLSELEQLRRSAMR   ScRunx1
81   ELRNASAVMKNQVARFNDLRFVGRSGRGKSFTLTITVFTNPPQVATYHRAIKVTVDGPREPRRFSDRLSDLGRIPHPSMR   HsRUNX2
81   ELRNASAVMKNQVARFNDLRFVGRSGRGKSFTLTITVLTNPPQVATYHRAIKVTVDGPREPRRFPERLSDLGRIPHPSMR   GgRunx2
81   ELRNASAVMKNQVARFNDLRFVGRSGRGKSFTLTITVFTNPPQVATYHRAIKVTVDGPREPRRFSERLSELERLRQTTMR   DrRunx2a
79   ELRNASGVMKNQVARFNDLRFVGRSGRGKSFTLTITVFTNPPQVATYHRAIKVTVDGPREPRRFSDRLSELERIRQTTMR   DrRunx2b
81   ELRNASAVMKNQVARFNDLRFVGRSGRGKSFTLTITVFTNPPQVATYHRAIKVTVDGPREPRRFSERLSELERLRQTAMR   CmRunx2
81   ELRNASAVMKNQVARFNDLRFVGRSGRGKSFTLTITVFTNPPQVATYHRAIKVTVDGPREPRRFSERLSELERLRQTAMR   ScRunx2
81   ELRNASAVMKNQVARFNDLRFVGRSGRGKSFTLTITVFTNPTQVATYHRAIKVTVDGPREPRRFPDRFGDLERLR---MR   HsRUNX3
81   ELRNASAVMKNQVARFNDLRFVGRSGRGKSFTLTITVFTNPTQVATYHRAIKVTVDGPREPRRFADRYGELERLRQS-MR   GgRunx3
81   ELRNASAVMKNQVARFNDLRFVGRSGRGKSFTLTITVFTGPPQVATYHRAIKVTVDGPREPRRFSDRLSDIERFQRASLR   DrRunx3
81   ELRNASAVMKNAVARFNDLRFVGRSGRGKSFTLTITVFTSPPQVATYHRAIKVTVDGPREPRRFSDRLSELERYRQTAMR   CmRunx3
81   ELRNASAVMKNTVARFNDLRFVGRSGRGKSFTLTITVFTSPPQVATYHRAIKVTVDGPREPRRFSDRLSELERYRQTTMR   ScRunx3
81   ELRNASAVMKNQVARFNDLRFVGRSGRGKSFTLTITVFTSPPQVATYHRAIKVTVDGPREPRRFPERLCELEQFRRSGMR   LjRunxA
81   ELRNASAVIKNQVARFNDLRFVGRSGRGKSFTLTITVFSNPPQVATYHRAIKVTVDGPREPRRFPDRLCDLDQLRRPTVR   MgRunxA
81   ELRNATAVMKQQVARFNDLRFVGRSGRGKSFTLTITVFTSPPQVATYHRAIKVTVDGPREPRIFSERLSELEHLRRTALR   LjRunxB
81   ELRNTRAVTKERVARFNDLRFVGRSGRGKSLTLTITVFTRPPQVATYQRAIKVTVDGPREPRRFTD----LEHYCRSSIR   MgRunxB
81   ELRNASAVMKGHVARFNDLRFVGRSGRGKSLNLTITVFTSPPQVATYQRAIKVTVDGPREPRRFPERL----------LQ   LjRunxC
81   ELRNASATMKGCVARFNDLRFLGRSGRGKSFNLTITIFSSPPQVATYQRAIKITVDGPREPRRFPEGFH-LESIRRNPTY   CiRunt
75   ELRNNQAVMKNQVARFNDLRFVGRSGRGKSFTLTITVFTSPPQVATYHRAIKVTVDGPREPRRFSERLSELERLRHSHIA   BfRunt
68   ELRNATAVMKNQVARFNDLRFVGRSGRGKTFSLTITVKTEPPQVATYCRAIKVTVDGPREPRRFPDRISDLDGFR-SRMR   NvRunx

161  VSPPNPRASLFNPQPQSQMTAPDLTAFLPSISD-----PRMHFTYSPTPVTSGYLPPPQAQGGPFQSYQFSMVGGERSRI   HsRUNX1
161  VSPPNPRASLFNPQPQSQIGASDLTAFLPSISD-----PRMHFTYTPTPVSSGYLPPPQAQGSPFQSYQFSMIGGDRSRI   GgRunx1
155  CSPPNTRPTLFGSPAHSQIHCPELTAFLPSLPDGRFSDPRVPFTYTPTPVTSAYLPPAQAQAGAFQSYQFSMMAGERSRI   DrRunx1
161  GSPPNPRHTLFNPQAHSQIVASDLTAFLPPLSDSRFPDPRMHFAYTPTPVTNGYLPPPQGQGGHFQSYQFSMMGGDRSRI   CmRunx1
161  LSPPNPRPSLFNPQAQSQIGASDLTAFLPSLSDGRFTDPRMHFPYTPTPVTTGYLPPPQGQTGPFQSYQFSMMGGDRSRI   ScRunx1
161  VGVQNPRPSLFNPQGQSQIGASELGPFISSLTESRFSNPRMHFTYTP-PVTSGYLPPPQSQSGPFQSYQFPMVGGDRSRM   HsRUNX2
161  VGVQSPRPSLFNPQGQSQIGASELGPFISSLTESRFSNPRMHFTYTP-PVTSGYLPPPQNQSGPFQSYQFPMVGGDRSRM   GgRunx2
161  VAVQSPRPSLFNPQGQTQIGTSELSPFLSSLTDSRFPSPRMHFTYTPTPVTSGYLPPPQNQSGPFQSYQFPMVGGDRSRM   DrRunx2a
159  VTVQTPRPSLYTPQGQTQIGSTDLSPFFSSLTESRFSSPRMHFTYTPTPVTTGYLPPPQSQSGPFQSYQFSMVGGERSRM   DrRunx2b
161  VGAQSPRHSLFSSQGQAQIGASDLGAFLSSLTDSRFSNPRMHFTYTPTPVTSGYLPPPTNQSGHFQSYQFSMVGGERSRM   CmRunx2
161  VGAQSPRPSLFSPQATSQIGATDFH-FLSSLTESRFPNPRMHFTYTPTPVTTGYLPPPPNQSGPFQSYQFSMVGGERSRM   ScRunx2
158  VTPPSPRGSLFSSQPQTPIGTSELNPFLPTLTESRFPDPRMHFPYSATPSGTSYLPPPQNQSGPFQSYQFSMVGGDRSRM   HsRUNX3
160  VTPPSPRGSIFGSQAQTPIGTSELSPFLPTLTETRFSDPRMHFPYTATPSATGYLPPPQNQSGPFQSYQFSMVGGERSRM   GgRunx3
161  MNPGTTRPH-YSPSSTTQITSTDLFQQLSPLTAPRFSDSHMHFTYSANPSSTGYLPPPQNQNSHFQSYQFSMIGGDRSRM   DrRunx3
161  VGPPSPRPQLFSSSAQTQMGSSDLSPFLSSLADSRFADPRVHFTYSATPPTTGYLPPPQNQGGTFQSYQFSMVAGERSRM   CmRunx3
161  VGPPSPRP-LFSPQAQTQMGTSDISPFLSSLTESRFSDPRMNFTYATTPSTGGYLPPPQNQSGPFQSYQFSMVGGERSRM   ScRunx3
161  VPPAAPRASLFNAQPQPQMASSELGAFLSSLPEGRFGDPRVHFAYTPGAASSAHLPPPQGQGSAFQSYQFPPPVGEERAT   LjRunxA
161  LSPGGSRAPLFNAQPQIQLGPGELGALLSSLSDSRFSDPRMHFHYPTAPTSAAHLPPPQNQVPPFQSYQFPMVSGERSRG   MgRunxA
161  VGAPALRPPLYNAQTDGQ-GGSELGPFLSYLPDSRNTDPRMHLSVYPTQVGAGYLPPPSHQSSPF-AYSLSVMGDERQTG   LjRunxB
157  HGTPTSGHQ-LNPSSHYTITGTEPGTCIPSLPDSHYGDSRLHPLYPAPPPGAGYLPPPNHQGSPYQSYQLSIIGEDRPTM   MgRunxB
151  LGPVTPTHDLFNPSRM---GLHHDGQF---LPD-----PRLHFAYPHLPAPPPYLPPPHAPSSAYQGYQLSLVSDQRSGS   LjRunxC
160  QGQSGTGGWALTPSQHAAAGGNGISPPFSEVHLGRFGNRSFVPGYDSSLPAGGYLYPANGNGGNSSDISSTMSGGARDNL   CiRunt
154  ---PAIRTPLHHPPPSAYTVISQRFPGDLRFSDPRLSDPRLLTAYSSGPTTT-MSPPGPRQLGNYSSYQYPILGGSGTSI   BfRunt
147  DPTPNPLQPNFSPLAEF-FQPTALQPPMPVLPH-VYPDARFQFQFRSTPMNNGYRSQPNMPQTTYQAMSFPMTMMSQSQG   NvRunx

236  LPPCTNASLPNQSDVVEAEGSHSNEAVWRPY   HsRUNX1
236  HPPCTNASLPNQSDVVEAEGSHSNEAVWRPY   GgRunx1
235  L-PCTNASLPNQSEVVEAEGSHSSEAVWRPY   DrRunx1
241  LPPCTNASLPNQNEVGETNGSHSNEGVWRPY   CmRunx1
241  LPPCTNASLPNQSDVVDADGSHSNEAVWRPY   ScRunx1
240  LPPCTTTSLPNQNDGVDADGSHSSESVWRPY   HsRUNX2
240  LPPCTTTSLPNQSDGVEADGSHSSESVWRPY   GgRunx2
241  LPPCTSASLPNQTDGGEADGSHSSESVWRPY   DrRunx2a
239  MPPCTSASLPVQADGVEGDGSHSNEGVWRPY   DrRunx2b
241  L-PCTSASLPNQNDGVDADGSHSSESVWRPY   CmRunx2
240  LPPCTSASLPNQNDGVDADGSHSNESVWRPY   ScRunx2
238  LASCTSSALGGQSDGVEADGSHSNEAVWRPY   HsRUNX3
240  LSSCTSASLGNQNDGVDADGSHSNESVWRPY   GgRunx3
240  LTSCTAAGLGNQSDGVDADGSHSNESVWRPY   DrRunx3
241  LPSCTSASLANQNDGVDPNGSQSNESVWRPY   CmRunx3
240  LPSCTSASLANQNDVVDADGSQSNESVWRPY   ScRunx3
241  APL-----LPSAHQGHDPEGSQGSEAVWRPY   LjRunxA
240  --------LPGTGE-TDAGTGHRSETVWRPY   MgRunxA
239  VGVCVGAGLSSPDGADGGDGSSSSESVWRPY   LjRunxB
236  GAPRLVVPLSSSDGGVEGDGSNENETVWRPY   MgRunxB
220  VGAC--------AEGTESDGSCPSEAVWRPY   LjRunxC
240  INSAVGLHLEAVEAGQRDNMDHHSEDVWRPY   CiRunt
231  LAATSNSQLPAQEKSQPGNGNHGNETVWRPY   BfRunt
225  LNSFTGFPEPPEGVGLELDHNHNGKGVWRPY   NvRunx
```
